# Recruitment of rare *3*-grams at functional sites: Is this a mechanism for increasing enzyme specificity?

**DOI:** 10.1186/1471-2105-8-226

**Published:** 2007-06-28

**Authors:** Dror Tobi, Ivet Bahar

**Affiliations:** 1 Department of Computational Biology, School of Medicine, University of Pittsburgh, Pittsburgh PA 15261, USA

## Abstract

**Background:**

A wealth of unannotated and functionally unknown protein sequences has accumulated in recent years with rapid progresses in sequence genomics, giving rise to ever increasing demands for developing methods to efficiently assess functional sites. Sequence and structure conservations have traditionally been the major criteria adopted in various algorithms to identify functional sites. Here, we focus on the distributions of the 20^3 ^different types of *3*-grams (or triplets of sequentially contiguous amino acid) in the entire space of sequences accumulated to date in the UniProt database, and focus in particular on the rare *3*-grams distinguished by their high entropy-based information content.

**Results:**

Comparison of the UniProt distributions with those observed near/at the active sites on a non-redundant dataset of 59 enzyme/ligand complexes shows that the active sites preferentially recruit *3*-grams distinguished by their low frequency in the UniProt. Three cases, Src kinase, hemoglobin, and tyrosyl-tRNA synthetase, are discussed in details to illustrate the biological significance of the results.

**Conclusion:**

The results suggest that recruitment of rare *3*-grams may be an efficient mechanism for increasing specificity at functional sites. Rareness/scarcity emerges as a feature that may assist in identifying key sites for proteins function, providing information complementary to that derived from sequence alignments. In addition it provides us (for the first time) with a means of identifying potentially functional sites from sequence information alone, when sequence conservation properties are not available.

## Background

Proteins perform a variety of biological functions, the efficiency and specificity of which are determined to a large extent by their intrinsic sequence-structure properties. Many biophysical and biochemical activities such as binding specific substrates, catalysis, or allosteric responses involve particular sequence motifs at functional sites. Identification of functional sites, or understanding sequence-to-function mapping, in general, has been a major goal in computational molecular biology and bioinformatics. With rapid accumulation of genome scale sequence data there is an ever increasing need for efficient assessment of potential functional sites.

Among different criteria adopted to identify functional sites, sequence conservation has probably been the most widely used, based on the fact that functional residues are often conserved across all or the majority of members in a given family of proteins. Algorithms developed for detecting conservation patterns are traditionally based on multiple sequence alignments [1-6]. Other algorithms use a combination of sequence and structure comparisons provided that structural information is available, which usually yield relatively higher predictive abilities [7-11]. A set of criteria was proposed by Thornton and coworkers for detecting catalytic residues, which was successfully implemented in an algorithm for identifying active sites [9]. Other strategies include pattern matching such as TESS [12], FFF [13] and SPASM [14] that locate functional sites by detecting small three-dimensional motifs. Proteins dynamics has been shown to be another property that can be advantageously examined to detect functional sites [15]. We have recently shown that the global hinge centers of enzymes co-localize with their catalytic sites, pointing to the importance of coupling between mechanics and chemistry for enzymatic activity [16]. In addition, analyzing patterns of communication and shortest paths in protein structures modeled as networks has proven useful in identifying allosteric sites [17,18].

In the present study, we propose another property that can serve as a criterion for detecting functionally important sites: *scarcity *or *rareness *in the space of sequence motifs. Motifs composed of three sequential amino acids (*3*-grams) are considered here as the shortest, yet distinctive, sequence fragments that can provide statistically significant information. The choice of *3*-grams in the following work is based on preliminary studies as well as our previous work [19] that showed that *1*-grams and *2*-grams do not confer enough specificity while there is no major difference in the performance of *3*-grams and *4*-grams (due to the fact that some strong signals characteristic of *3*-grams may be overlooked upon examination of *4*-grams), while *3*-grams lend themselves to more accurate statistics. A given *3*-gram will be termed '*rare' *if its probabilistic occurrence in the UniProt (Universal Protein Resource) [20] is significantly lower than that expected from an unbiased distribution, as will be explained in quantitative details below.

A notable feature emerging from this study is the propensity of proteins' active sites to populate rare *sequence *motif, akin to the notion that rare *structural *motifs co-localize with functionally important sites [21-23]. This selectivity suggests that enzymes tend to recruit such distinctive/rare sequences at their active sites to increase their specificity, consistent with the higher information content associated with rare events. We illustrate the functional importance of such rare *3*-grams in three proteins, C-Src kinase, hemoglobin, and tyrosyl-tRNA synthetase.

## Results and discussion

### Distributions of *3*-grams

**Figure 1:**
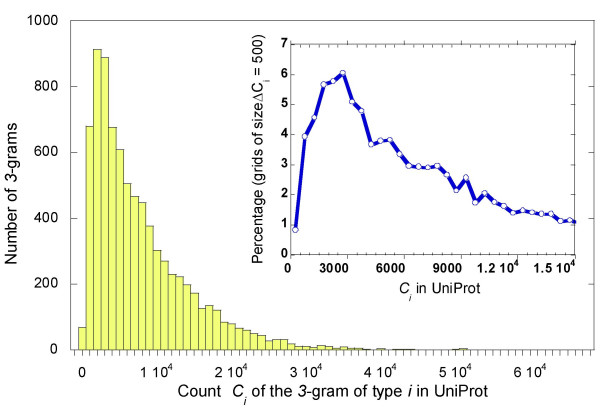
***Distribution of 3-grams in UniProt***. Histogram of the counts *C*_*i *_for all types (1 ≤ *i *≤ 20^3^) of *3*-grams, shown for grids of size Δ*C*_*i *_= 10^3^. The mean <*C_i_*> and standard deviation σ_C _are 7,822 and 6,682, respectively. The inset shows the portion of the curve for *C*_*i*_≤ 15,000 in more detail. The ordinate is the percentage of *3*-grams within intervals of Δ*C*_*i *_= 500. The peak (6.07%) occurs at the interval 2500 ≤ *C_i_*≤ 3000. *3*-grams that are one standard deviation away from the mean towards lower counts are termed 'rare' *3*-grams. Their counts are lower than [<*C_i_*> - σ_C_] = 1,140. There is a total of 480 such *3*-grams (i.e. 6% of all the 8,000 types of 3-grams), and their cumulative frequency of occurrence evaluated from the ratio of their total count to *C*_*tot *_is 0.595%.

A given protein sequence of *N *residues is viewed as a collection of *N – n *+ 1 overlapping words of *n *letters, termed *n*-grams (also called *n*-tuples), composed of *n *contiguous amino acids along the sequence. For each protein, we consider all *3*-grams (*n *= 3) or triplets, by sliding a window of three amino acids along the sequence, thus leading to a total of *(N – 2) *triplets. The natural frequency of a given *3*-gram of type *i *(1 ≤ *i *≤ 20^3^) is defined as

piuni=Ci∑Cj
 MathType@MTEF@5@5@+=feaafiart1ev1aaatCvAUfKttLearuWrP9MDH5MBPbIqV92AaeXatLxBI9gBaebbnrfifHhDYfgasaacH8akY=wiFfYdH8Gipec8Eeeu0xXdbba9frFj0=OqFfea0dXdd9vqai=hGuQ8kuc9pgc9s8qqaq=dirpe0xb9q8qiLsFr0=vr0=vr0dc8meaabaqaciaacaGaaeqabaqabeGadaaakeaacqWGWbaCdaqhaaWcbaGaemyAaKgabaGaemyDauNaemOBa4MaemyAaKgaaOGaeyypa0ZaaSaaaeaacqWGdbWqdaWgaaWcbaGaemyAaKgabeaaaOqaamaaqaeabaGaem4qam0aaSbaaSqaaiabdQgaQbqabaaabeqab0GaeyyeIuoaaaaaaa@3C31@

where *C*_*i *_is the number of occurrence in the UniProt, and the summation is performed over all 20^3 ^types of *3*-grams in the 172,233 sequences compiled as of March 1, 2005, which contain a total number *C*_*tot *_= 62,256,868 of *3*-grams. Each of the 20^3 ^distinctive types is represented in UniProt. Their counts vary in the range 170 <*C*_*i *_< 67,264, with the lower and upper bounds corresponding to the *3*-grams MCW and AAA, respectively. Figure 1 displays the distribution of the counts *C*_*i*_. The inset shows a closer view of the percentage of *3*-grams types whose UniProt counts fall in successive grids of size Δ *C*_*i *_= 500, for the range *C*_*i*_≤ 1.5 10^4^. We note that less than 1% of the 20^3 ^types have UniProt counts *C*_*i *_lower than 500, and 14.97% has *C*_*i *_< 2,000. Rare *3*-grams are defined as those in the lower end of the histogram, located one standard deviation away from the mean. They comprise 6.0% of all 3-gram *types*, and 0.595% of all 3-grams *counts *in the UniProt (see Figure 1 caption).

The unbiased probability of a given *3*-gram is *p*_0 _= 1/20^3 ^= 0.000125, and the corresponding count is *C*_0 _= *C*_*tot*_/20^3 ^= 7782 in the UniProt. The *3*-grams whose count is lower than this value by a factor of *f *= 2 compose 10% of all the counts *C*_*tot *_in UniProt, and those lower by *f *= 3 amount to 4% of *C*_*tot*_.

### Scarcity scores and enhancement factors

**Figure 2:**
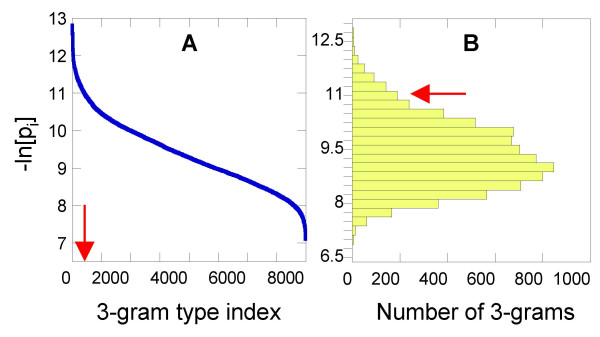
***Scarcity Scores***. **(A) **Scarcity scores (eq 2) plotted as a function of *3*-gram index, sorted in descending order. **(B) **Histogram of scarcity scores. The abscissa shows the number of 3-grams having scarcity scores values lying in successive ranges of size 0.25 shown along the ordinate. The peak occurs at siuni
 MathType@MTEF@5@5@+=feaafiart1ev1aaatCvAUfKttLearuWrP9MDH5MBPbIqV92AaeXatLxBI9gBaebbnrfifHhDYfgasaacH8akY=wiFfYdH8Gipec8Eeeu0xXdbba9frFj0=OqFfea0dXdd9vqai=hGuQ8kuc9pgc9s8qqaq=dirpe0xb9q8qiLsFr0=vr0=vr0dc8meaabaqaciaacaGaaeqabaqabeGadaaakeaacqWGZbWCdaqhaaWcbaGaemyAaKgabaGaemyDauNaemOBa4MaemyAaKgaaaaa@33D6@ = 9.0 ± 0.125. Unique *3*-grams (Figure 1) lie in the range siuni
 MathType@MTEF@5@5@+=feaafiart1ev1aaatCvAUfKttLearuWrP9MDH5MBPbIqV92AaeXatLxBI9gBaebbnrfifHhDYfgasaacH8akY=wiFfYdH8Gipec8Eeeu0xXdbba9frFj0=OqFfea0dXdd9vqai=hGuQ8kuc9pgc9s8qqaq=dirpe0xb9q8qiLsFr0=vr0=vr0dc8meaabaqaciaacaGaaeqabaqabeGadaaakeaacqWGZbWCdaqhaaWcbaGaemyAaKgabaGaemyDauNaemOBa4MaemyAaKgaaaaa@33D6@ > 10.908, or *i *< 480, indicated by the arrow, and their observed cumulative frequency is piuni
 MathType@MTEF@5@5@+=feaafiart1ev1aaatCvAUfKttLearuWrP9MDH5MBPbIqV92AaeXatLxBI9gBaebbnrfifHhDYfgasaacH8akY=wiFfYdH8Gipec8Eeeu0xXdbba9frFj0=OqFfea0dXdd9vqai=hGuQ8kuc9pgc9s8qqaq=dirpe0xb9q8qiLsFr0=vr0=vr0dc8meaabaqaciaacaGaaeqabaqabeGadaaakeaacqWGWbaCdaqhaaWcbaGaemyAaKgabaGaemyDauNaemOBa4MaemyAaKgaaaaa@33D0@ < 1.83 10^-5^.

The frequency piuni
 MathType@MTEF@5@5@+=feaafiart1ev1aaatCvAUfKttLearuWrP9MDH5MBPbIqV92AaeXatLxBI9gBaebbnrfifHhDYfgasaacH8akY=wiFfYdH8Gipec8Eeeu0xXdbba9frFj0=OqFfea0dXdd9vqai=hGuQ8kuc9pgc9s8qqaq=dirpe0xb9q8qiLsFr0=vr0=vr0dc8meaabaqaciaacaGaaeqabaqabeGadaaakeaacqWGWbaCdaqhaaWcbaGaemyAaKgabaGaemyDauNaemOBa4MaemyAaKgaaaaa@33D0@ given by eq (1) will also be termed the 'observed' or 'natural' probability. We will associate with each *3*-gram type a *scarcity score *given by

siuni=−ln⁡[piuni]
 MathType@MTEF@5@5@+=feaafiart1ev1aaatCvAUfKttLearuWrP9MDH5MBPbIqV92AaeXatLxBI9gBaebbnrfifHhDYfgasaacH8akY=wiFfYdH8Gipec8Eeeu0xXdbba9frFj0=OqFfea0dXdd9vqai=hGuQ8kuc9pgc9s8qqaq=dirpe0xb9q8qiLsFr0=vr0=vr0dc8meaabaqaciaacaGaaeqabaqabeGadaaakeaacqWGZbWCdaqhaaWcbaGaemyAaKgabaGaemyDauNaemOBa4MaemyAaKgaaOGaeyypa0JaeyOeI0IagiiBaWMaeiOBa4Maei4waSLaemiCaa3aa0baaSqaaiabdMgaPbqaaiabdwha1jabd6gaUjabdMgaPbaakiabc2faDbaa@4247@

which provides a measure of the information content each *3*-gram carries. The distribution of scarcity scores is given in Figure 2. They vary in the range 6.83 ≤ siuni
 MathType@MTEF@5@5@+=feaafiart1ev1aaatCvAUfKttLearuWrP9MDH5MBPbIqV92AaeXatLxBI9gBaebbnrfifHhDYfgasaacH8akY=wiFfYdH8Gipec8Eeeu0xXdbba9frFj0=OqFfea0dXdd9vqai=hGuQ8kuc9pgc9s8qqaq=dirpe0xb9q8qiLsFr0=vr0=vr0dc8meaabaqaciaacaGaaeqabaqabeGadaaakeaacqWGZbWCdaqhaaWcbaGaemyAaKgabaGaemyDauNaemOBa4MaemyAaKgaaaaa@33D6@ ≤ 12.81. Panel A displays the scores sorted in descending order, plotted against *3*-gram type index, and panel B displays their histogram (distribution among the 20^3 ^types), based on grids of size Δsiuni
 MathType@MTEF@5@5@+=feaafiart1ev1aaatCvAUfKttLearuWrP9MDH5MBPbIqV92AaeXatLxBI9gBaebbnrfifHhDYfgasaacH8akY=wiFfYdH8Gipec8Eeeu0xXdbba9frFj0=OqFfea0dXdd9vqai=hGuQ8kuc9pgc9s8qqaq=dirpe0xb9q8qiLsFr0=vr0=vr0dc8meaabaqaciaacaGaaeqabaqabeGadaaakeaacqWGZbWCdaqhaaWcbaGaemyAaKgabaGaemyDauNaemOBa4MaemyAaKgaaaaa@33D6@ = 0.25. The arrows display the threshold adopted for defining rare 3-grams.

The 'expected' probability of *3*-gram *i *= {XYZ} on the other hand, based on the natural occurrences of individual amino acids, assuming the probabilistic occurrence of amino acids to be independent of their sequential neighbors, is

piexp=p0Xp0Yp0Z
 MathType@MTEF@5@5@+=feaafiart1ev1aaatCvAUfKttLearuWrP9MDH5MBPbIqV92AaeXatLxBI9gBaebbnrfifHhDYfgasaacH8akY=wiFfYdH8Gipec8Eeeu0xXdbba9frFj0=OqFfea0dXdd9vqai=hGuQ8kuc9pgc9s8qqaq=dirpe0xb9q8qiLsFr0=vr0=vr0dc8meaabaqaciaacaGaaeqabaqabeGadaaakeaacqWGWbaCdaqhaaWcbaGaemyAaKgabaacbiGae8xzauMae8hEaGNae8hCaahaaOGaeyypa0JaemiCaa3aaWbaaSqabeaacqaIWaamaaGccqWGybawcqWGWbaCdaahaaWcbeqaaiabicdaWaaakiabdMfazjabdchaWnaaCaaaleqabaGaeGimaadaaOGaemOwaOfaaa@403C@

where *p*^0 ^*X *is the natural frequency of amino acid of type X (for details see Table S1 in Additional file 1). The enrichment in a given *3*-gram count will be described by the enhancement factor

ai=piuni/piexp
 MathType@MTEF@5@5@+=feaafiart1ev1aaatCvAUfKttLearuWrP9MDH5MBPbIqV92AaeXatLxBI9gBaebbnrfifHhDYfgasaacH8akY=wiFfYdH8Gipec8Eeeu0xXdbba9frFj0=OqFfea0dXdd9vqai=hGuQ8kuc9pgc9s8qqaq=dirpe0xb9q8qiLsFr0=vr0=vr0dc8meaabaqaciaacaGaaeqabaqabeGadaaakeaacqWGHbqydaWgaaWcbaGaemyAaKgabeaakiabg2da9iabdchaWnaaDaaaleaacqWGPbqAaeaacqWG1bqDcqWGUbGBcqWGPbqAaaGccqGGVaWlcqWGWbaCdaqhaaWcbaGaemyAaKgabaacbiGae8xzauMae8hEaGNae8hCaahaaaaa@3FC7@

**Figure 3:**
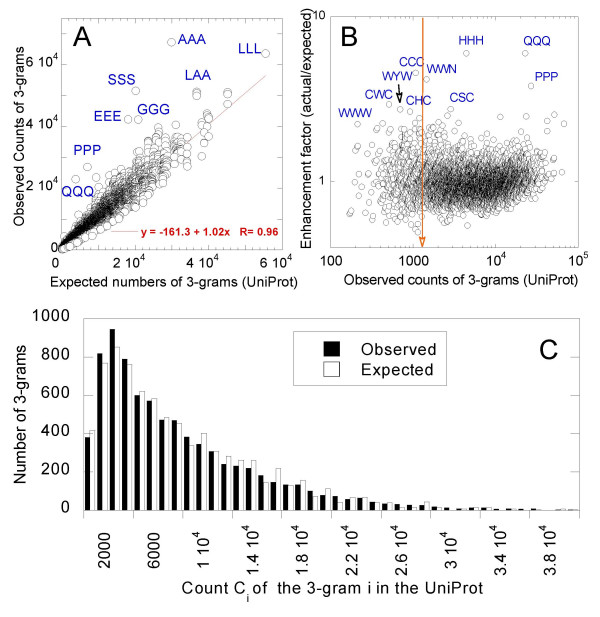
***Observed vs expected counts of 3-grams***. **(A) **Observed counts (*C*_*i*_) are directly retrieved for all 20^3 ^types of *3*-grams from the UniProt. Expected counts are based on the natural frequencies of individual amino acids (eq 3). Observed counts (*C*_*i*_) usually match the expected ones (slope of best fitting line is 1.02), although particular *3*-grams (labeled) are enhanced. The correlation coefficient between observed and expected counts is 0.96. The enrichment *a*_*i *_in particular *3*-grams is shown in panel B. The observed counts are plotted in a log-log scale, to provide a clearer view of the subset of unique *3*-grams (to the left of the arrow). **(B) **Comparison of observed histogram of 3-grams (Figure 1) and that expected from the independent frequencies of occurrences of amino acids in each triplet. The two histograms are significantly different (χ^2^, *P < 0.001)*.

Figure 3A compares the observed and expected counts of *3*-grams. While the slope gives an average enhancement factor <*a*_*i*_> = 1.02 with a correlation coefficient of 0.96, a more detailed examination of expected and observed *3*-grams Figure 3C shows that the two distributions exhibit statistically significant departure (χ^2^, *P < 0.001)*. The expected histogram is more broadly distributed than that observed. Some *3*-grams that show substantial departures from their expected frequencies (i.e. enhancement factor *a*_*i*_≠ 1) are labeled in panel B. Among over-represented rare *3*-grams we observe CCC, WWN, WYW, CWC, CHC and WWW. We also note that AAA and LLL are distinguished by their high piuni
 MathType@MTEF@5@5@+=feaafiart1ev1aaatCvAUfKttLearuWrP9MDH5MBPbIqV92AaeXatLxBI9gBaebbnrfifHhDYfgasaacH8akY=wiFfYdH8Gipec8Eeeu0xXdbba9frFj0=OqFfea0dXdd9vqai=hGuQ8kuc9pgc9s8qqaq=dirpe0xb9q8qiLsFr0=vr0=vr0dc8meaabaqaciaacaGaaeqabaqabeGadaaakeaacqWGWbaCdaqhaaWcbaGaemyAaKgabaGaemyDauNaemOBa4MaemyAaKgaaaaa@33D0@, and HHH and QQQ by their enhancement *a*_*i *_≥ 6. The HHH enrichment may, however, arise from tagged histidines, rather than reflecting a natural preference.

The present approach permits us to assign a scarcity score to each amino acid in a given sequence without recourse to sequence alignment. In the calculations below we have performed similar analyses for protein families, where the occurrence of rare *3*-grams at specific locations where examined with regard to their conservation properties. Given an alignment of homologues sequences, an average scarcity score was assigned to each position *j *along a sequence *k*, based on the minimum value, puni|jk=min{p    auni,p    bunip    cuni}jk
 MathType@MTEF@5@5@+=feaafiart1ev1aaatCvAUfKttLearuWrP9MDH5MBPbIqV92AaeXatLxBI9gBamXvP5wqSXMqHnxAJn0BKvguHDwzZbqegyvzYrwyUfgarqqtubsr4rNCHbGeaGqiA8vkIkVAFgIELiFeLkFeLk=iY=Hhbbf9v8qqaqFr0xc9pk0xbba9q8WqFfeaY=biLkVcLq=JHqVepeea0=as0db9vqpepesP0xe9Fve9Fve9GapdbaqaaeGacaGaaiaabeqaamqadiabaaGcbaGaemiCaa3aaWbaaSqabeaacqWG1bqDcqWGUbGBcqWGPbqAaaGccqGG8baFdaWgaaWcbaGaemOAaOMaem4AaSgabeaakiabg2da9GWaciaa=1gacaWFPbGaa8NBaiabcUha7jabdchaWnaaDaaaleaacqqGGaaicqqGGaaicqqGGaaicqqGGaaicqqGHbqyaeaacqqG1bqDcqqGUbGBcqqGPbqAaaGccqGGSaalcqWGWbaCdaqhaaWcbaGaeeiiaaIaeeiiaaIaeeiiaaIaeeiiaaIaeeOyaigabaGaeeyDauNaeeOBa4MaeeyAaKgaaOGaemiCaa3aa0baaSqaaiabbccaGiabbccaGiabbccaGiabbccaGiabbogaJbqaaiabbwha1jabb6gaUjabbMgaPbaakiabc2ha9naaBaaaleaacqWGQbGAcqWGRbWAaeqaaaaa@706E@, of the three consecutive *3*-grams of types (*a*, *b*, *c*) that contain residue *j*, using

<sjuni>=−ln⁡[<pjuni>]=−ln⁡[m−1Σkpuni|jk]
 MathType@MTEF@5@5@+=feaafiart1ev1aaatCvAUfKttLearuWrP9MDH5MBPbIqV92AaeXatLxBI9gBaebbnrfifHhDYfgasaacH8akY=wiFfYdH8Gipec8Eeeu0xXdbba9frFj0=OqFfea0dXdd9vqai=hGuQ8kuc9pgc9s8qqaq=dirpe0xb9q8qiLsFr0=vr0=vr0dc8meaabaqaciaacaGaaeqabaqabeGadaaakeaacqGH8aapcqWGZbWCdaqhaaWcbaGaemOAaOgabaGaemyDauNaemOBa4MaemyAaKgaaOGaeyOpa4Jaeyypa0JaeyOeI0IagiiBaWMaeiOBa4Maei4waSLaeyipaWJaemiCaa3aa0baaSqaaiabdQgaQbqaaiabdwha1jabd6gaUjabdMgaPbaakiabg6da+iabc2faDjabg2da9iabgkHiTiGbcYgaSjabc6gaUjabcUfaBjabd2gaTnaaCaaaleqabaGaeyOeI0IaeGymaedaaOGaeu4Odm1aaSbaaSqaaiabdUgaRbqabaGccqWGWbaCdaahaaWcbeqaaiabdwha1jabd6gaUjabdMgaPbaakiabcYha8naaBaaaleaacqWGQbGAcqWGRbWAaeqaaOGaeiyxa0faaa@5E71@

where the summation is performed over the homologous sequences (1 ≤ *k *≤ *m*) in the alignment.

### Proteins tend recruit rare *3*-grams at their active sites

The biological significance of rare *3*-grams has been examined by analyzing the results with regard to the dataset of 59 non-redundant enzyme/ligand complexes compiled by Gutteridge and Thornton [24], shortly referred to as the GT dataset (Table 1). Two non-overlapping subsets of *3*-grams were retrieved from the GT dataset. The first is composed of all *3*-grams located at the active sites, and the second includes all other residues. The two subsets comprise 3,514 and 17,450 *3*-grams, respectively. Active site *3*-grams are defined as those containing at least one heavy atom located within 5Å from the ligand.

**Table 1:** GT (Gutteridge-Thornton, 2005)^24 ^dataset of 59 enzymes*

1cib	1kbqAC	6aldA	1n2uA	1o7n	3apr
8acn	1de6A	1d6sA	1cml	1oaf	6cel
1a16	3daa	1fs5A	1eexABC	1pnf	1jkxA
1b66A	1b57	1jcr	2tlx	1dz8A	2dhc
1b74	1e7y	1gai	1eixAB	1ibv	4reqAB
1m7yA	1dzt	1ggf	1lee	1cq1A	2tsc
1lruA	1k9sAD	1gm7AB	1erm	1e8gA	1oneA
1dag	1uae	1gkm	1hduA	1lmt	1ra2
1hm2A	1esd	1o6g	1ma0	1bd0	1ldm
1esw	1dud	1e9gA	1mka	1g49B	

* The table lists the PDB code and chains used. Care was taken not to double count *3*-grams in cases where the active site is located at the interface of two identical subunits.

**Figure 4:**
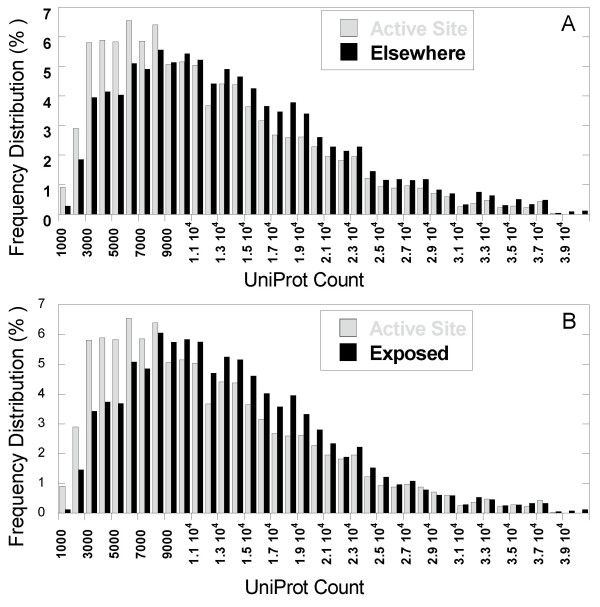
***Comparison of natural occurrences of 3-grams***. Two subsets of *3*-grams are compared **(A) **those located at (1) active sites of enzymes vs. (2) other positions and **(B) **those located at (1) active sites of enzymes vs. (2) exposed ones (see Methods). The 3-grams in an ensemble of 59 non-redundant enzyme/ligand complexes [24] were analyzed with regard to their natural frequencies of occurrence in the UniProt. The abscissa shows the number of occurrence of the examined *3*-grams in UniProt, divided into intervals of size Δ*Ci = *1,000, and the ordinate shows the fraction of *3*-grams fall in the successive count intervals, for the two subsets of 3-grams: active sites (gray) and others/exposed (black). Comparison of the two histograms reveals the higher proportion of rare *3*-grams in the active sites. In particular the range *C*_*i *_< 1,140 (rare *3*-grams) shows a propensity for active site *3*-grams that is enhanced by a factor of 2.67 compared to other *3*-grams and by a factor of 5.4 compared to exposed *3*-grams. Chi square test resulted with P << 0.001 for both histograms indicating the statistical significance of the differences.

The UniProt frequencies of these two groups of *3*-grams yielded the histograms displayed in Figure 4A. To obtain these histograms, the ensemble of *3*-grams in the respective subsets 1 and 2 of active site residues (gray) and other residues (black) have been organized into groups of different UniProt frequencies, and the counts of *3*-grams in each group have been determined. The abscissa shows the ranges of UniProt counts, divided into intervals of Δ *C*_*i *_= 1000, starting from *C*_*i*_≤ 1,000 (labeled 1000), and the ordinate represents the population (or probability) of each group. Not all 20^3 ^types of *3*-grams were represented in this set of 59 enzymes.

Comparison of the two histograms immediately reveals the tendency of the active site *3*-grams to populate the lower UniProt counts. For example, let us consider the rare *3*-grams (with *C*_*i *_< 1,140 or siuni
 MathType@MTEF@5@5@+=feaafiart1ev1aaatCvAUfKttLearuWrP9MDH5MBPbIqV92AaeXatLxBI9gBaebbnrfifHhDYfgasaacH8akY=wiFfYdH8Gipec8Eeeu0xXdbba9frFj0=OqFfea0dXdd9vqai=hGuQ8kuc9pgc9s8qqaq=dirpe0xb9q8qiLsFr0=vr0=vr0dc8meaabaqaciaacaGaaeqabaqabeGadaaakeaacqWGZbWCdaqhaaWcbaGaemyAaKgabaGaemyDauNaemOBa4MaemyAaKgaaaaa@33D6@ > 10,908; see Methods and Figure 2). 11.67% of active site *3*-grams fall in this range, whereas the percentage drops to 4.35% when *3*-grams at other regions are considered. Therefore, rare *3*-grams are over-represented in the active sites by a factor of 2.67. The difference between the two distributions shown in panel A is statistically significant (χ^2^, *P << 0.001)*.

Similarly, we compared the histogram obtained for the subset of active site *3*-grams with that calculated for solvent-exposed *3*-grams (see Methods). Figure 4B shows the histograms for active site (gray) and exposed (black) residues. The active sites are again distinguished by the propensity of rare *3*-grams. Rare 3-grams are 5.4 times more frequent at active sites compared to exposed regions (χ^2^, *P << 0.001) *. The difference between the two histograms is more pronounced than that observed in panel A.

**Figure 5:**
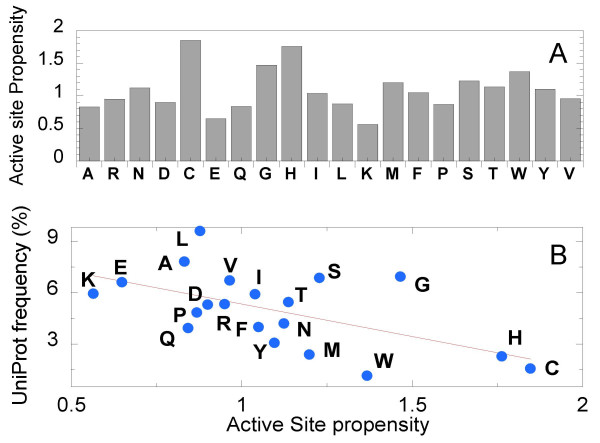
***Active site propensity of amino acids and comparison with their natural frequencies***. **(A) **For each type of amino acids, the frequencies of occurrence in the active and non-active sites were calculated. The ratio between them is defined as the active site propensity (ordinate). **(B) **Natural (UniProt) frequency of occurrence vs. active site propensity for each type of amino acid. Less frequent amino acids tend to have a higher active site propensity, and *vice versa*, in accord with the histograms in Figure 4. This effect becomes more pronounced at the level of *3*-grams.

Next, we examined the identity of *3*-grams at the active sites, and focused in particular on the rare *3*-grams that are recruited. Given that not all of the 20^3 ^types of *3*-grams were represented in the GT dataset, not all types of *3*-grams might be either seen at the active sites. We first analyzed the amino acid composition of active sites. For each amino acid, the probabilities of being located at the active site versus elsewhere were calculated. The ratio between these two probabilities, termed the *active site propensity*, is shown in Figure 5A. Four amino acids, Cys, His, Gly, and Trp, are distinguished by their high active site propensities. This is in agreement with previous work [25] where His and Cys were shown to have the highest propensity for serving as catalytic residues via their side chains, while Gly has the highest propensity of catalyzing reactions via its main chain. We also note that, in the present study, Arg and Glu exhibit relatively low tendencies to locate at active sites, while these two amino acids were reported by Bartlett *et al *[25] to have relatively high catalytic propensities. This difference presumably arises from the different definitions adopted for active sites in the two studies: we refer to all amino acids located in the neighborhood of the ligand, while Bartlett *et al *[25] referred to residues directly involved in *catalytic *activity.

Cys and His, two residues that have the highest active site propensity (both in terms of their ligand-binding and catalytic activities) are among the least frequent amino acids in terms of their natural occurrence. Figure 5A shows the relation between the frequencies of amino acids in UniProt and their active site propensities. While the data are scattered, there is a discernible anti-correlation between the two sets of data, consistent with Figure 4; rare amino acids have a higher tendency to be in active sites. Clearly this preference becomes more pronounced when *3*-grams, as opposed to monograms are examined, as a can be seen from the skewed distribution of the frequency of active site residues towards lower counts in Figure 4.

Rare *3*-grams may arise from three possibilities: (1) these may be words in which the individual letters have low *natural *frequencies themselves (i.e. *a*_*i *_≈ 1), (2) these may be rare 'words', i.e. rare combinations of particular amino acids (*a*_*i *_<< 1); or (3) these may be rare despite their enhancement (*a*_*i *_>>1) due to the extremely low natural frequencies of the individual amino acids that compose them. Counterparts of Figure 3 plotted for the active site *3*-grams and other *3*-grams showed that both groups fall in the first category (*a*_*i *_≈ 1). Their observed counts plotted against the expected ones yield a slope of 1.075 ± 0.008. The rare *3*-grams belonging to the two groups, on the other hand, exhibited a slight enhancement of *a*_*i *_= 1.11 and 1.07, for active site and other residues, respectively. Thus, *3*-grams recruited at active sites are usually combinations of residues which already have relatively low natural frequencies of occurrence. Yet, particular *3*-grams exhibit significant departure from their expected occurrence probabilities, e.g. SSS, GGG, RRR, GSG (over-represented), and LEL (under-represented). See Table S2 and S3 in Additional files for details. Among rare *3*-grams at active sites, also exhibiting a large enhancement in the UniProt (case 3, above), we distinguish WWH and HCW.

### Scarce *3*-grams in the GT dataset

Scarcity scores were calculated for all *3*-grams in each of the 59 proteins in the GT dataset. The postulate that rare *3*-grams (above a threshold scarcity score) preferentially locate in active sites was examined for a series of threshold scores. For each enzyme, the rates of true positives (TP) and false positives (FP) were evaluated as a function of threshold scarcity scores, TP rates being defined as the fraction of active site 3-grams that exhibit scarcity score above the threshold value. Table 2 shows the average ratios between the rates of TPs and FPs for seven different thresholds. The uppermost threshold (s_max _= 11) shows more than threefold enhancement in the rate of TPs relative to FPs, while the discriminative power of the scarcity score vanishes as we lower the threshold. A detailed list of TP and FP rates along with the top ranking *3*-grams are provided for each enzyme in the Additional files Table S4.

**Table 2:** Results for the GT dataset

**Scarcity Score Threshold**	TP/FP*
11.0	3.152
10.5	1.936
10.0	1.543
9.5	1.432
9.0	1.353
8.5	1.139
8.0	1.041

* The average ratio between true positives (TP) rates and false positives (FP) rates for the dataset of 59 enzymes

### Illustrative results for three cases

The functional role of rare *3*-grams at proteins' functional sites is illustrated here for three test cases, a Src tyrosine kinase, hemoglobin and a tyrosyl-tRNA synthetase. For each case, a set of homologous proteins has been compiled and aligned, and the scarcity score <sjuni
 MathType@MTEF@5@5@+=feaafiart1ev1aaatCvAUfKttLearuWrP9MDH5MBPbIqV92AaeXatLxBI9gBaebbnrfifHhDYfgasaacH8akY=wiFfYdH8Gipec8Eeeu0xXdbba9frFj0=OqFfea0dXdd9vqai=hGuQ8kuc9pgc9s8qqaq=dirpe0xb9q8qiLsFr0=vr0=vr0dc8meaabaqaciaacaGaaeqabaqabeGadaaakeaacqWGZbWCdaqhaaWcbaGaemOAaOgabaGaemyDauNaemOBa4MaemyAaKgaaaaa@33D8@> of amino acid(s) at each position has been calculated. The analysis reveals that stretches of rare amino acids are involved in functional roles even beyond ligand binding sites.

#### Src kinase

**Figure 6:**
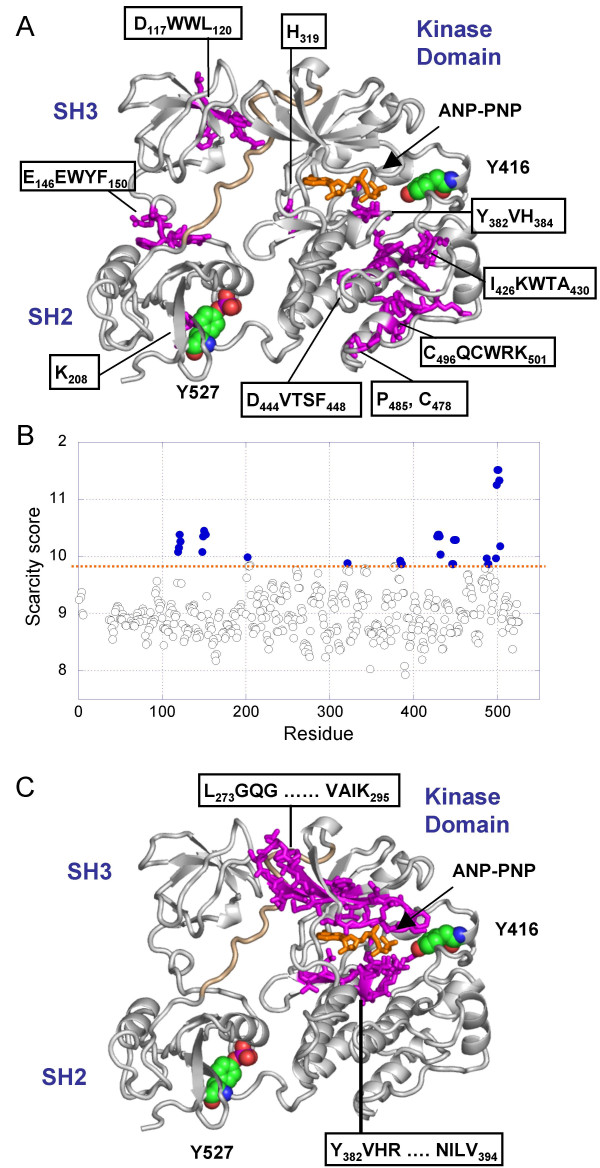
***(A) Identification of c-Src unique n-grams***. c-Src inactive conformation (PDB:2SRC [58]) is shown as a ribbon diagram (white), the AMP-PNP as a stick model (orange), the phosphotyrosines Y416 and Y527 in space-filling representation, and SH2-kinase domain linker (wheat). The most unique residues are shown as stick model along with their side chains. All ribbon diagrams are drawn using Pymol [59]. **(B) C-Src scarcity scores**. 111 human Src homologous proteins (E value < 6.10^-57^) were used in calculations. The abscissa represents the residue index and the ordinate the scarcity score. Filled circles refer to top ranking 32 amino acids colored in panel A, most of which are functional and conserved despite being highly rare in the UniProt. **(C) PROSITE motifs**. Scanning the c-Src sequence against the PROSITE database reveals two recognized motifs (magenta) corresponding to the ATP binding site and the catalytic loop.

The Src family of non-receptor tyrosine kinases is a member of the tyrosine kinases superfamily and plays an important role in cell differentiation, proliferation and survival [26]. C-Src, a member of Src protein kinase family, contains two peptide binding modules (SH3 and SH2 domains), a catalytic tyrosine kinase domain (composed of N- and C-lobes) and a C-terminal regulatory tail (for a review see [27]) (Figure 6A). Its phosphorylation at activation loop residue Y527 results in intramolecular interaction between the SH2 domain and the phosphorylated C-terminal tail, triggering a transition into an inactive conformation with the SH2 and SH3 domains moving away from the catalytic site clamping the latter in a strained conformation. Dephosphorylation of Y527 and phosphorylation of Y416, on the other hand, activate the kinase via movement of the activation loop and opening of the catalytic cleft between the N- and the C- lobes of the kinase domain.

Figure 6B shows the scarcity scores <sjuni
 MathType@MTEF@5@5@+=feaafiart1ev1aaatCvAUfKttLearuWrP9MDH5MBPbIqV92AaeXatLxBI9gBaebbnrfifHhDYfgasaacH8akY=wiFfYdH8Gipec8Eeeu0xXdbba9frFj0=OqFfea0dXdd9vqai=hGuQ8kuc9pgc9s8qqaq=dirpe0xb9q8qiLsFr0=vr0=vr0dc8meaabaqaciaacaGaaeqabaqabeGadaaakeaacqWGZbWCdaqhaaWcbaGaemOAaOgabaGaemyDauNaemOBa4MaemyAaKgaaaaa@33D8@> as a function of residue number along the c-Src sequence, based on UniProt counts of *3*-grams, averaged over the set of *m *aligned homologous sequences (eq 5). Six *n*-grams (3 ≤ *n *≤ 6) composed of contiguous *3*-grams are distinguished by their high scores, D_117_WWL_120_, E_146_EWYF_150_, Y_382_VH_384_, I_426_KWTA_430_, D_444_VWSF_448_, and C_496_QCWRK_501_, in addition to individual residues H319, K200, C478 and P485.

D_117_WWL_120 _is located in the SH3 domain, and interacts with the linker that connects the SH2 and kinase domains when the kinase adopts its inactive conformation (Figure 6A). The SH3-linker interactions have been pointed out to serve as an independent mode of regulation for Hck, a Src family member [28]. In addition, Trp118, at the ligand-binding site of SH3 [29], was found to be essential to the stability of the SH3-VSL12 complex [30].

The second stretch of amino acids, E_146_EWYF_150_, coincides with the SH2-SH3 linker which was shown by Kuriyan and coworkers [31] to work as an inducible "snap-lock" that clamps the SH2 and SH3 domains upon phosphorylation of Y572. Mutations of residues in the linker have been shown to lead to a constitutive activation of c-Src [31].

The third stretch, Y_382_VH_384_, forms the Src catalytic loop. Y_382 _takes part in key interactions that stabilize the kinase domain in the inactive conformation [32] and H_384 _belongs to the conserved HRD motif of the catalytic loop. Lys200 at SH2 domain interface interacts with the C-terminal regulatory tail.

The other three stretches of amino acids I_426_KWTA_430_, D_444_VWSF_448_, and C_496_QCWRK_501 _form a cluster located at and beneath the substrate binding region (Figure 6A). The portion Q_497_CWR_500 _is composed of two overlapping *3*-grams that have the highest scarcity scores in the protein. Notably, the central residues in these stretches, W499, C498, **W428**, **W446**, are highly or fully (bold) conserved, lending support to their critical role.

A systematic analysis of amino acid conservation conducted for c-Src protein family members demonstrated that amino acids distinguished by their high scarcity scores tend to be conserved among the family members (see Conclusion), despite their being rare in the UniProt. Conserved sites distinguished by their scarcity include, in addition to the four residues listed above, W148, K427, T429, F448, S447, A430, H319, **H384**, C487, **D444 **and V445. See Additional files Table S5 for details.

Figure 6C shows the two motifs derived for Src kinase from PROSITE database [33]. The first is the stretch of twenty two amino acids L_273_GQGCFG ... GTTRVAIK_295 _corresponding to the ATP binding region and the second is the stretch Y_382_VHRDLRAANILV_394 _corresponding to the catalytic loop. Except for the *3*-gram Y_382_VH_384 _identified in both panels A and C, the 3-grams deduced from scarcity score and PROSITE analyses appear to provide complementary information.

#### Hemoglobin

**Figure 7:**
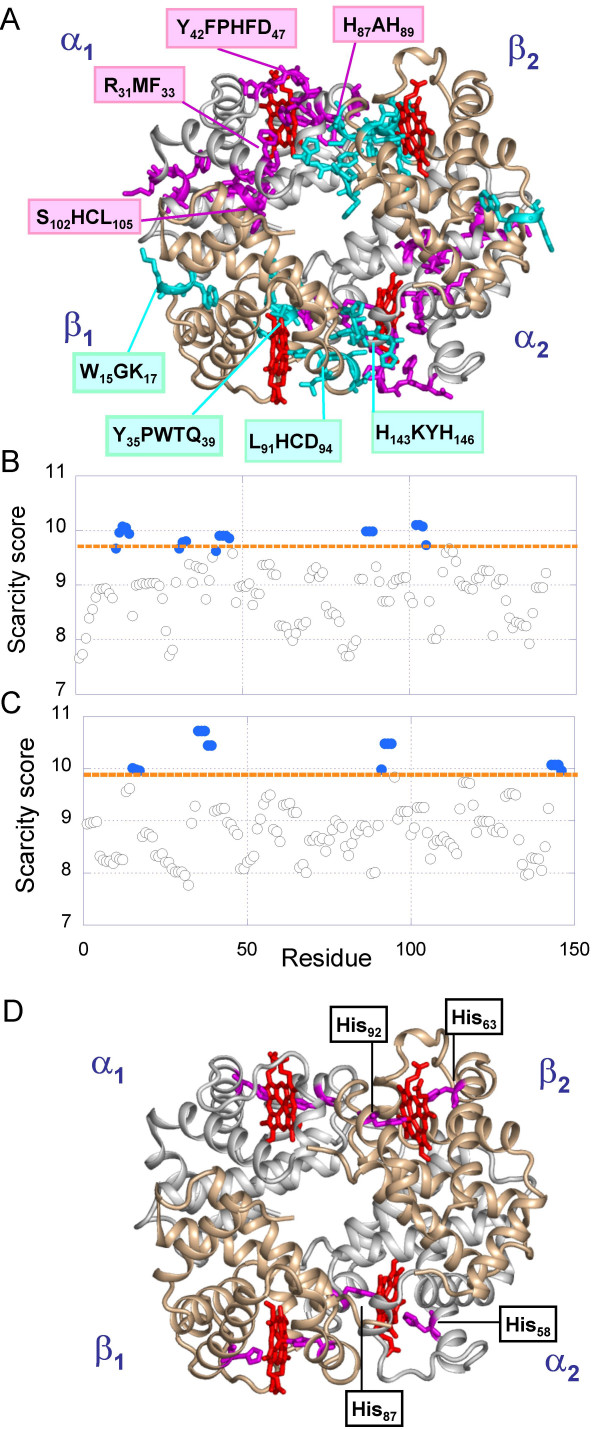
***(A) Identification of unique n-grams in hemoglobin***. The deoxy hemoglobin structure (PDB:1A3N [60]) is shown in Panel A. The tetramer is composed of two α-subunits, (white) two β-subunits (wheat) and four heme groups (red). The most unique amino acids are colored magenta (α-subunit) and cyan (β-subunit). (**B, C**)** Scarcity scores for the respective α- and β-subunits**. The scores are based on 138 and 224 homologous sequences (E value < 10^-57^) retrieved for a- and b-subunits, respectively. The residues colored in panel A have scarcity scores (based on 3-grams to which they belong; see eq 5) above the threshold indicated by the orange dashed line. **(D) PROSITE motifs**. Two histidines (magenta) are identified at the heme binding sites using PROSITE for each subunit.

Hemoglobin (Hb) is a tetramer composed of two α- and two β-subunits (α_1_, α_2_, β_1 _and β_2_) organized in two dimers, α_1_β_1 _and α_2_β_2_, symmetrically positioned around a central water-filled cavity (Figure 7A). Each subunit has a heme that binds oxygen. The oxygenation of Hb is cooperative, i.e. binding of a first O_2 _enhances the O_2 _affinity of the other subunits. Following the MWC model [34], Hb exists in two conformations in rapid equilibrium: the T state with low affinity for oxygen binding and the R state with high affinity. The intrinsic oxygen binding affinity of the tetramer is nearly 300-fold lower than that of its free α_1_β_1 _dimer [35], implying that the interactions at the dimer-dimer interface interfere with substrate binding unless relieved by a conformational switch.

The <sjuni
 MathType@MTEF@5@5@+=feaafiart1ev1aaatCvAUfKttLearuWrP9MDH5MBPbIqV92AaeXatLxBI9gBaebbnrfifHhDYfgasaacH8akY=wiFfYdH8Gipec8Eeeu0xXdbba9frFj0=OqFfea0dXdd9vqai=hGuQ8kuc9pgc9s8qqaq=dirpe0xb9q8qiLsFr0=vr0=vr0dc8meaabaqaciaacaGaaeqabaqabeGadaaakeaacqWGZbWCdaqhaaWcbaGaemOAaOgabaGaemyDauNaemOBa4MaemyAaKgaaaaa@33D8@> profiles for subunits α and β are presented in Figure 7B and 7C, respectively. Five stretches of residues are distinguished by their high scarcity scores in subunit α : α A_12_AWGK_16_, α R_31_MF_33_, α Y_42_FPHF_47_, α H_87_AH_89_, and α S_102_HCL_105_, colored magenta in panel A. In subunit β, β W_15_GK_17_, β Y_35_PWTQ_39_, β L_91_HCD_94 _and β H_143_KYH_146 _(cyan) emerge as rare sequences.

Notably, all of these stretches of amino acids distinguished by their high scarcity score assume functional roles in Hb. α R_31_MF_35 _and α Y_42_FPHFD_47 _are directly involved in binding the heme group. The mutant R31S results in abnormal hemoglobin – Hb Prato [36]. α Tyr42 forms an inter-subunit hydrogen bond with β Asp99 across the dimer-dimer interface in the T state of hemoglobin but not on the R state. Kavanaugh *et al *[37] showed that this hydrogen bond and other interactions associated with the side chain of α Tyr42 make a major contribution to the stability of the T state. α H_87_AH_89 _corresponds to the heme binding site, with α His87 coordinating the Fe atom. The Fe-Nε bonds between the heme group and α His87 or β His92 are essential for the cooperativity of hemoglobin [38]. α His103 is located inside a central cavity lined with excess of positively charged ionizable groups. It has been suggested [39] and experimentally validated [40] that the mutual repulsion of these ionizable groups increases the oxygen affinity by raising the free energy of the T state. Using network analysis, del Sol *et al*. confirmed that α His103 plays an important role in allosteric communication [17]. We also note the critical position of α His103 at the α_1_β_1 _interface with α Ser102 pointing toward the heme binding pocket. Among the residues participating in rare sequences in subunit β, in addition to the heme-binding β H92, we note β K17, the substitution of which by Asn has been reported to lead to abnormal Hb J Amiens [41]. The segment β Y_35_PWTQ_39 _is located at the convergence of the α_1_β_1 _and α_2_β_2 _interfaces and is important for allostery. Mutations of β 37W (e.g. β W37E and β W37G) have been shown to lead to tetrameric Hb with functional properties similar to those of the αβ dimers; that is, high oxygen affinity with no cooperativity [42,43]. Finally, the *4*-gram β H_143_KYH_146 _at the C-terminal end of subunit β and in particular H_143 _is a binding site for allosteric effectors [44,45].

In addition to the above experimental evidence, we note that using the Gaussian network model we have shown that two segments, α F36-H45 and β T87-β N102, play a key role in the allosteric transition of Hb from constrained (T form) to flexible (or relaxed) (R2 form) state [46]; these two regions include the so-called switch region [47]. The first stretch partially overlaps with α Y_42_FPHF_46 _and the second contains β L_91_HCD_94_. These results lend further support to the critical role of the rare *n*-grams, and in particular β His92, in propagating allosteric signals. Finally, we note that out of 37 rare residues identified in Hb, 14 are fully conserved (entropy S = 0), and 17 highly conserved (S < 0.75; see Additional files Table S6 for more details).

Upon scanning the hemoglobin sequence against the PROSITE database (Figure 7D) four histidines are retrieved, two (α His58 and α His87) on α-subunits, and two (β His63 and β His92) on β-subunits. As mentioned above, α His87 and β His92, also detected among the rare 3-grams, coordinate the Fe atom of the heme group, while α His58 and β His63 are located at the same region across the heme plane.

#### Tyrosyl-tRNA synthetase

**Figure 8:**
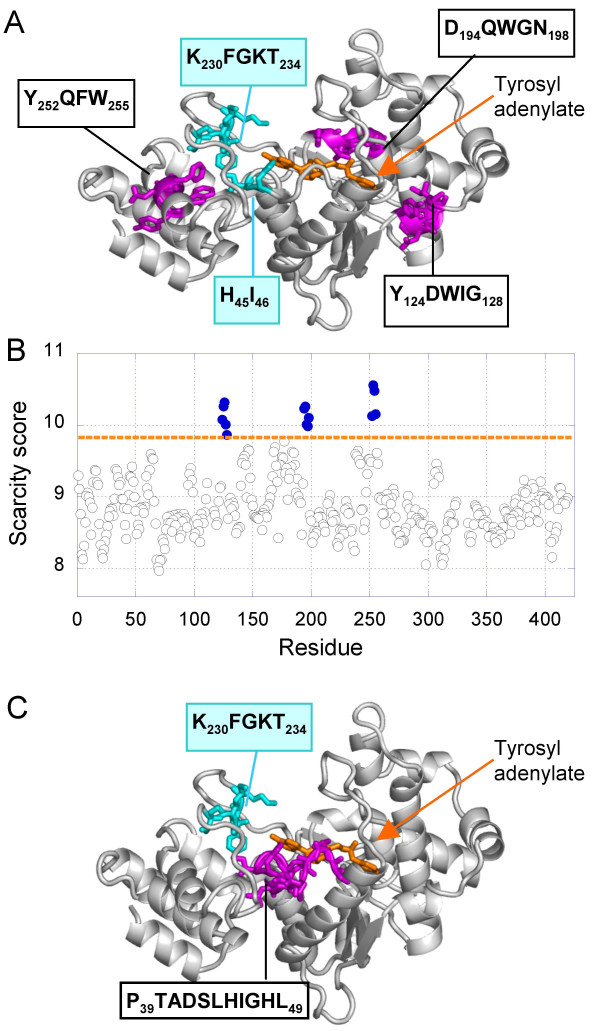
***(A) Identification of tyrosyl tRNA synthetase unique n-grams***. Tyrosyl -tRNA synthetase (PDB:3TS1 [48]) is shown (white) in complex with tyrosyl adenylate intermediate (orange). The pair H_45_I_46 _(cyan) of the catalytic His, the residues K_230_FGKT_234 _of the mobile loop (cyan) and the three most unique *n*-grams (magenta) are shown as sticks.**(B) Scarcity score **of amino acids. 24 homologous (E value < 10^-57^) of *Bacillus stearothermophilus *were used to calculate the average uniqueness score. Three unique *n*-grams (filled circles) are distinguished. **(C) PROSITE motifs**. The loop containing the catalytic His at the binding site (magenta) is identified using PROSITE.

Tyrosyl-tRNA synthetase catalyzes the attachment of tyrosine to its cognate tRNA after activation of the tyrosine via formation of tyrosyl adenylate. Its structure has been solved in complexed form with an inhibitor (Figure 8A) that mimics the binding of the activated tyrosine (tyrosyl adenylate) in the active site [48]. The calculated <sjuni
 MathType@MTEF@5@5@+=feaafiart1ev1aaatCvAUfKttLearuWrP9MDH5MBPbIqV92AaeXatLxBI9gBaebbnrfifHhDYfgasaacH8akY=wiFfYdH8Gipec8Eeeu0xXdbba9frFj0=OqFfea0dXdd9vqai=hGuQ8kuc9pgc9s8qqaq=dirpe0xb9q8qiLsFr0=vr0=vr0dc8meaabaqaciaacaGaaeqabaqabeGadaaakeaacqWGZbWCdaqhaaWcbaGaemOAaOgabaGaemyDauNaemOBa4MaemyAaKgaaaaa@33D8@> profile shown in Figure 8B reveals three *n*-grams distinguished by their uniqueness: Y_124_DWIG_128_, D_194_QWGN_198_, and Y_252_QFW_255 _(Figure 8A, magenta). The former two are located at the substrate binding site. The latter forms contacts with the conserved motif HIGH that comprises the catalytic His45 [49] and with the amino acids F231, G232, and T234 of the K_230_FGKT_234 _mobile loop (Figure 8A, cyan). This loop was found to stabilize the transition state in the activation reaction of tyrosyl by ATP [50]. We note that **W126**, **D194, Q195**, W196, G197, **N198**, Y252 and **Q253 **are fully (bold) or highly conserved. See Table S7 in the Additional files for more details. PROSITE scanning reveals the P_39_TADSLHIGHL_49 _stretch of amino acids at the active site, which includes the catalytic His45 (Figure 8C).

### Results in the absence of sequence alignment

In the above three examples the scarcity score was calculated by averaging *p*^*uni*^|_*jk *_over a set of homologous proteins. However, in principle, scarcity score calculations can be equally performed for a single sequence as the ingredient is the UniProt frequencies for triplets along the sequence. The alignments are simply used to magnify (increase the accuracy of) the signals that could, otherwise, be extracted from single sequence analysis. As shown in the figures S1–S3 in the Additional files, the sjuni
 MathType@MTEF@5@5@+=feaafiart1ev1aaatCvAUfKttLearuWrP9MDH5MBPbIqV92AaeXatLxBI9gBaebbnrfifHhDYfgasaacH8akY=wiFfYdH8Gipec8Eeeu0xXdbba9frFj0=OqFfea0dXdd9vqai=hGuQ8kuc9pgc9s8qqaq=dirpe0xb9q8qiLsFr0=vr0=vr0dc8meaabaqaciaacaGaaeqabaqabeGadaaakeaacqWGZbWCdaqhaaWcbaGaemOAaOgabaGaemyDauNaemOBa4MaemyAaKgaaaaa@33D8@ profiles based on single sequences closely approximate those derived from multiple sequence alignments. For hemoglobin subunit α, for example, six stretches of amino acids are distinguished by their high scarcity scores: W_14_GK_16_, R_31_MF_33_, F_43_PHF_46_, D_75_MPN_78_, H_87_AH_89_, S_102_HCL_105_, in accord with the results presented above. The only stretch of amino acids that differs from the above analysis is D_75_MPN_78_. Thus, when sequence conservation algorithms fail, the *3*-gram analysis of single chains can be advantageously resorted to for identifying potentially functional sites.

## Conclusion

The present study shows that relatively rare sequence motifs are preferentially recruited at enzymes' active sites. The probabilistic occurrence of rare *3*-grams at active sites is higher than that of other 3-grams by a factor of more than 2.6 and higher that of exposed *3*-grams by a factor of 5.4 according to the results obtained for the GT dataset of 59 enzymes (Figure 4). Detailed analysis of each enzyme in this dataset demonstrated that a scarcity score threshold of 11.0, permits us to identify 3-grams located at/near active sites, with a TP rate more than three times larger than the FP rate. These results were obtained from the sequence of the individual enzymes without recourse to sequence alignment or any amino acid conservation information.

The preferential selection of rare *3*-grams at functional sites may not be a property of enzymes, exclusively, but that of proteins, in general, as the current application to hemoglobin suggests. We have illustrated and discussed here the functional importance of rare *3*-gram motifs for three proteins: c-Src, hemoglobin and tyrosyl-tRNA synthetase.

**Figure 9:**
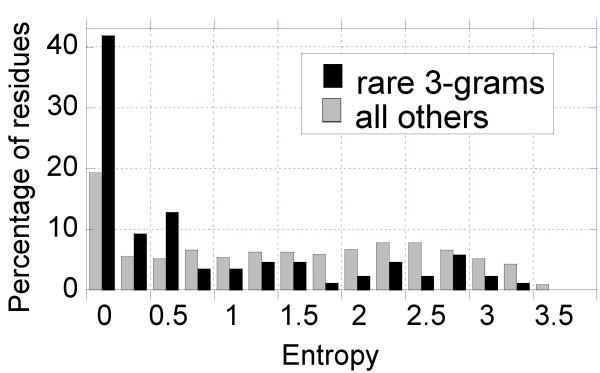
***Histogram of Shannon entropies***. for amino acids participating in rare 3-grams (black) and other amino acids (dashed), based on the family memberships in the examined three cases (Src, Hb and Tyr tRNA synthetase). The abscissa shows the percentages of amino acids corresponding to bins of size Δ S = 0.25. We note the high propensity of rare residues in the range S < 0.25 corresponding to highest conservation.

As a further assessment of the functional importance of rare *n*-grams, we examined the conservation of these *n*-grams among the members of the three families of proteins on which we focused. Figure 9 displays the results, reported in terms of the Shannon entropies [51], for two groups of residues: (i) the rare residues distinguished by their high scarcity scores (above the threshold values indicated in Figures 6B, 7B, C and 8B), and (ii) all other residues. The respective numbers of residues in the two sets are 83 and 1240. The histograms (percentages) are displayed for bins of size 0.25. We note that unique residues exhibit a stronger tendency to be conserved (despite their low counts in the UniProt) among the members of the examined families, lending further support to their functional importance (see Additional files for details).

While our analysis is purely *sequence-based*, it appears to be conceptually in accord with two *structure-based *studies that indicate that rare structural motifs correlate with proteins functional sites. Petock *et al *[22] showed that 9-residue fragments of rare backbone conformations often form parts of ligand-binding sites, protein-protein interactions, and domain-domain contacts. Likewise, Novotny and Kleywegt [23] found that, even thought left-handed helices are rare, when they do occur, they are structurally or functionally significant. The present analysis shows that not only structural motifs, but sequence motifs as well, tend to be highly distinctive at active sites. This selection of rare sequence motifs seems to be driven by specificity requirements. Notably, the rarest amino acids (Cys, His, Trp and Met; Figure 5B) have unique functional properties: Cys and Met are sulfur-containing amino acids and are sensitive to oxidation. Likewise, His is unique as its protonation depends on pH, and Trp is distinguished by its bulky size and significant contribution to stabilization of hydrophobic cores or interfaces. It has been suggested that proteins may even sustain a decrease in their stability in order to form effective functional sites [21,22]. In accord with the above notion, these highly specific residues, Trp, His, Met, and Cys, may be used by enzymes for precise functional purposes, their specificity being amplified in *3*-grams where two or more of these amino acids are juxtaposed.

Identification of proteins' functional sites using computational tools is a major challenge in computational biology in the post-genomic era [52,53]. Sequence and structure conservation are the major criteria used by current algorithms to identify functional important sites. Identification of functional sites for orphan proteins (for which no homologous protein exists) is beyond the scope of algorithms that use conservation as major criterion for identifying these sites. Notably, we repeated the scarcity score analysis by using only a single chain and we were still able to identify functionally important sites (see Additional files), demonstrating that the potentially functional sites of orphan proteins can also be assessed by our methodology, and that conservation and scarcity are two different features. We note that the average scarcity score of scarce *and *conserved residues will be higher than that of scarce and non-conserved residues, as the scarcity score is evaluated as an average over all aligned sequences. However, calculations repeated for single query sequences, as opposed to those averaged over multiple aligned sequences showed that such effects are negligibly small and the profile of scarcity scores for examined proteins are robust. In addition, we have compared the scarcity scores (based on UniProt frequencies) and the Shannon entropies at the corresponding amino acid positions. Computations for the three test cases revealed that residues participating in rare 3-grams (Tables S5–S7) are not necessarily conserved or *vice versa*. However, they do exhibit a higher tendency to be conserved compared to other amino acids, as illustrated in Figure 9. Therefore, it is conceivable that a combined approach exploiting both conservation and scarcity may enhance the score/signal associated with particular sites, thus increasing the ability to accurately identify functional motifs.

## Methods

### Datasets

Present calculations are based on the data extracted from UniProt [20] version 4.2. This database was chosen because it is manually curated with minimal level of redundancy and still comprehensive. Furthermore, UniProt contains entries from multiple organisms and it is therefore less susceptible to biases in *3*-gram distributions of specific species as noted by Ganpathiraju et al [54].

The biological implications of the *3*-grams distinguished by high scarcity scores were examined using the dataset of 59 enzyme/ligand complexes compiled by Gutteridge and Thornton [24] (GT dataset; see Table 1). We performed three case studies: C-Src kinase, hemoglobin subunits α and β, and tyrosyl-tRNA synthetase. Homologous sequences were retrieved in each case by a PBLAST search [55] of the SwissProt database. Sequences with *E*-values lower than 10^-57 ^were extracted, leading to *m *= 111 homologous sequences for c-Src, 138 and 224 sequences for hemoglobin α- and β-subunits, respectively, and 24 sequences for tyrosyl tRNA synthetase. The sequences were aligned with ClustalX using default parameters [56].

### Exposed *3*-grams

The Accessible surface area (ASA) of each amino acid in the GT database was calculated using the program NACCESS [57]. Amino acids were defined as exposed if their relative exposed surface are was > 20%. *3*-grams with two or more exposed amino acids were defined as exposed ones.

## Authors' contributions

DT did the calculations. DT and IB analyzed the results and wrote the manuscript. Both authors read and approved the final manuscript.

## Supplementary Material

Additional File 1 **Supplementary tables S1-S7**. Figures and material. Click here for file
