# Supplementary material for: Recruitment of rare 3-grams at functional sites: Is this a mechanism for increasing enzyme specificity?
Source: BMC Bioinformatics. 2007 Jun 28;8:226. doi: 10.1186/1471-2105-8-226 (PMC1950313; doi:10.1186/1471-2105-8-226)
Supplement: Additional File 1 — Supplementary tables S1-S7. Figures and material. [file 1471-2105-8-226-S1.doc]

**Supplementary Material**

**A. Additional Tables**

**Table S1: Amino acids frequencies (percentage) in the UniProt**

| **Amino Acid** | **UniProt**  **Frequency** |  | **Amino Acid** | **UniProt**  **Frequency** |
| --- | --- | --- | --- | --- |
| W | 1.15423 |  | R | 5.326978 |
| C | 1.558473 |  | T | 5.455844 |
| H | 2.279128 |  | I | 5.918905 |
| M | 2.379281 |  | K | 5.93725 |
| Y | 3.072935 |  | E | 6.621828 |
| Q | 3.944846 |  | V | 6.724865 |
| F | 4.004677 |  | S | 6.869617 |
| N | 4.200068 |  | G | 6.942564 |
| P | 4.838353 |  | A | 7.832152 |
| D | 5.314707 |  | L | 9.623299 |

**Table S2: Most enhanced 3-grams at active sites**

| **Type** | **UniProt**  **Counts (*)** | **Enhancement**  **factor** |  | **Type** | **UniProt**  **Counts** | **Enhancement**  **Factor (*a_i_*)** |
| --- | --- | --- | --- | --- | --- | --- |
| SSS | 51519 | 2.5527 |  | EKI | 22840 | 1.5766 |
| RRR | 23492 | 2.4965 |  | WCP | 852 | 1.5749 |
| WHH | 777 | 2.0831 |  | HGH | 3501 | 1.5595 |
| GGG | 42216 | 2.0265 |  | NNI | 10126 | 1.5578 |
| WRW | 804 | 1.8231 |  | HYT | 3704 | 1.5576 |
| FHP | 4978 | 1.8108 |  | YEC | 3070 | 1.5552 |
| RYW | 2118 | 1.8010 |  | CCL | 2259 | 1.5526 |
| LQQ | 15958 | 1.7117 |  | NYY | 3825 | 1.5492 |
| WWH | 317 | 1.6772 |  | HCW | 393 | 1.5412 |
| HFH | 2170 | 1.6757 |  | GSG | 31759 | 1.5407 |
| KKY | 11159 | 1.6549 |  | GKT | 21536 | 1.5383 |
| PAP | 18829 | 1.6496 |  | PSS | 21642 | 1.5225 |
| DYY | 5105 | 1.6341 |  | QQA | 11536 | 1.5203 |
| QQP | 7629 | 1.6277 |  | QRQ | 7823 | 1.5161 |
| YYN | 3930 | 1.5917 |  | PHP | 5004 | 1.5068 |
| SFW | 3145 | 1.5916 |  | RQQ | 7749 | 1.5017 |
| KNI | 14610 | 1.5899 |  | LKE | 35340 | 1.5004 |
| CHT | 1902 | 1.5771 |  |  |  |  |

(*) total counts in UniProt = 62,256,868

**Table S3: Least enhanced 3-grams at active sites**

| **Type** | **UniProt**  **Counts** | **Enhancement**  **factor** |  | **Type** | **UniProt**  **Counts** | **Enhancement**  **factor (*a_i_*)** |
| --- | --- | --- | --- | --- | --- | --- |
| EAN | 9456 | 0.69730 |  | NRG | 6452 | 0.66720 |
| LMY | 3050 | 0.69630 |  | FKF | 3952 | 0.66680 |
| FEL | 11047 | 0.69530 |  | EGP | 9203 | 0.66460 |
| ESA | 15287 | 0.68920 |  | CAK | 2985 | 0.66170 |
| AEC | 3461 | 0.68780 |  | KFA | 7657 | 0.66050 |
| KGA | 13806 | 0.68690 |  | KAP | 9249 | 0.66030 |
| RPK | 6521 | 0.68450 |  | NGP | 5778 | 0.65790 |
| HKG | 3992 | 0.68260 |  | REP | 6730 | 0.63340 |
| LKF | 9650 | 0.67740 |  | KGP | 7742 | 0.62360 |
| AMW | 905 | 0.67590 |  | WPI | 1270 | 0.61740 |
| AEP | 10466 | 0.67000 |  | KWP | 1067 | 0.51700 |

**Table S4: True Positive and False Positive rates for enzymes in GT dataset as a function of threshold scarcity score (*)**

| **Scarcity score** | **TP** | **FP** |  | **Scarcity score** | **TP** | **FP** |
| --- | --- | --- | --- | --- | --- | --- |
| -1cib- |  | |  | -1lruA- |  |  |
| 11.000 | 0.000 | 0.000 |  | 11.000 | 0.000 | 0.000 |
| 10.500 | 0.025 | 0.000 |  | 10.500 | 0.026 | 0.000 |
| 10.000 | 0.038 | 0.033 |  | 10.000 | 0.077 | 0.009 |
| 9.500 | 0.076 | 0.108 |  | 9.500 | 0.205 | 0.099 |
| 9.000 | 0.139 | 0.239 |  | 9.000 | 0.308 | 0.144 |
| 8.500 | 0.367 | 0.464 |  | 8.500 | 0.487 | 0.441 |
| 8.000 | 0.861 | 0.788 |  | 8.000 | 0.769 | 0.820 |
| TQWG *YHN* | | |  | MDH HEM CIQ | | |
| -8acn- |  |  |  | -1dag- |  |  |
| 11.000 | 0.019 | 0.003 |  | 11.000 | 0.000 | 0.000 |
| 10.500 | 0.019 | 0.008 |  | 10.500 | 0.018 | 0.000 |
| 10.000 | 0.115 | 0.042 |  | 10.000 | 0.055 | 0.029 |
| 9.500 | 0.327 | 0.134 |  | 9.500 | 0.109 | 0.137 |
| 9.000 | 0.577 | 0.244 |  | 9.000 | 0.182 | 0.302 |
| 8.500 | 0.788 | 0.495 |  | 8.500 | 0.473 | 0.547 |
| 8.000 | 0.962 | 0.829 |  | 8.000 | 0.818 | 0.763 |
| *CHY* *CNM* CDH | | |  | WFT IPW *HAM* | | |
| -1a16_- |  |  |  | -1hm2A- |  |  |
| 11.000 | 0.000 | 0.006 |  | 11.000 | 0.065 | 0.007 |
| 10.500 | 0.042 | 0.014 |  | 10.500 | 0.065 | 0.018 |
| 10.000 | 0.083 | 0.059 |  | 10.000 | 0.161 | 0.058 |
| 9.500 | 0.229 | 0.135 |  | 9.500 | 0.242 | 0.150 |
| 9.000 | 0.375 | 0.293 |  | 9.000 | 0.339 | 0.323 |
| 8.500 | 0.708 | 0.499 |  | 8.500 | 0.645 | 0.583 |
| 8.000 | 0.938 | 0.808 |  | 8.000 | 0.935 | 0.853 |
| *FWY* *CEM* SHW | | |  | WWH NWW WEW | | |
| -1b66A- |  |  |  | -1esw- |  |  |
| 11.000 | 0.000 | 0.000 |  | 11.000 | 0.023 | 0.005 |
| 10.500 | 0.000 | 0.011 |  | 10.500 | 0.046 | 0.038 |
| 10.000 | 0.100 | 0.046 |  | 10.000 | 0.149 | 0.082 |
| 9.500 | 0.267 | 0.126 |  | 9.500 | 0.322 | 0.147 |
| 9.000 | 0.500 | 0.287 |  | 9.000 | 0.448 | 0.275 |
| 8.500 | 0.867 | 0.414 |  | 8.500 | 0.713 | 0.477 |
| 8.000 | 1.000 | 0.782 |  | 8.000 | 0.862 | 0.755 |
| *YIW* CNN HNY | | |  | WKW *WFH* WAW | | |
| -1b74- |  |  |  | -1kbqAC- |  |  |
| 11.000 | 0.000 | 0.000 |  | 11.000 | 0.000 | 0.000 |
| 10.500 | 0.042 | 0.000 |  | 10.500 | 0.041 | 0.013 |
| 10.000 | 0.125 | 0.005 |  | 10.000 | 0.122 | 0.047 |
| 9.500 | 0.167 | 0.038 |  | 9.500 | 0.243 | 0.101 |
| 9.000 | 0.250 | 0.143 |  | 9.000 | 0.514 | 0.221 |
| 8.500 | 0.292 | 0.438 |  | 8.500 | 0.743 | 0.450 |
| 8.000 | 0.583 | 0.776 |  | 8.000 | 0.932 | 0.711 |
| CTH CNT *HNF* | | |  | HFC *WPI* QWF | | |

| **Scarcity score** | **TP** | **FP** |  | **Scarcity score** | **TP** | **FP** |
| --- | --- | --- | --- | --- | --- | --- |
| -3daa- |  |  |  | -1esd- |  |  |
| 11.000 | 0.000 | 0.000 |  | 11.000 | 0.000 | 0.011 |
| 10.500 | 0.000 | 0.000 |  | 10.500 | 0.000 | 0.015 |
| 10.000 | 0.045 | 0.060 |  | 10.000 | 0.000 | 0.042 |
| 9.500 | 0.091 | 0.136 |  | 9.500 | 0.105 | 0.107 |
| 9.000 | 0.250 | 0.307 |  | 9.000 | 0.368 | 0.211 |
| 8.500 | 0.523 | 0.608 |  | 8.500 | 0.579 | 0.513 |
| 8.000 | 0.864 | 0.864 |  | 8.000 | 0.684 | 0.793 |
| *CYE* IRW *WTR* | | |  | *WCF GWC HFW* | | |
| -1b57- |  |  |  | -1dud- |  |  |
| 11.000 | 0.000 | 0.000 |  | 11.000 | 0.000 | 0.000 |
| 10.500 | 0.017 | 0.004 |  | 10.500 | 0.000 | 0.000 |
| 10.000 | 0.086 | 0.038 |  | 10.000 | 0.000 | 0.019 |
| 9.500 | 0.293 | 0.103 |  | 9.500 | 0.182 | 0.087 |
| 9.000 | 0.483 | 0.244 |  | 9.000 | 0.455 | 0.221 |
| 8.500 | 0.647 | 0.506 |  | 8.500 | 0.500 | 0.481 |
| 8.000 | 0.871 | 0.835 |  | 8.000 | 0.864 | 0.808 |
| *HQM* DHC HCA | | |  | *HKH MMK* AMM | | |
| -1e7y- |  |  |  | -6aldA- |  |  |
| 11.000 | 0.000 | 0.000 |  | 11.000 | 0.000 | 0.003 |
| 10.500 | 0.013 | 0.006 |  | 10.500 | 0.000 | 0.014 |
| 10.000 | 0.053 | 0.039 |  | 10.000 | 0.000 | 0.066 |
| 9.500 | 0.133 | 0.096 |  | 9.500 | 0.000 | 0.149 |
| 9.000 | 0.320 | 0.261 |  | 9.000 | 0.107 | 0.308 |
| 8.500 | 0.467 | 0.584 |  | 8.500 | 0.357 | 0.505 |
| 8.000 | 0.773 | 0.882 |  | 8.000 | 0.643 | 0.820 |
| HTM *WKF* *WVF* | | |  | *WRC* *CQY* *HAC* | | |
| -1dzt- |  |  |  | -1d6sA- |  |  |
| 11.000 | 0.000 | 0.000 |  | 11.000 | 0.000 | 0.004 |
| 10.500 | 0.000 | 0.025 |  | 10.500 | 0.000 | 0.004 |
| 10.000 | 0.067 | 0.089 |  | 10.000 | 0.000 | 0.027 |
| 9.500 | 0.150 | 0.139 |  | 9.500 | 0.019 | 0.058 |
| 9.000 | 0.333 | 0.215 |  | 9.000 | 0.185 | 0.159 |
| 8.500 | 0.617 | 0.405 |  | 8.500 | 0.426 | 0.438 |
| 8.000 | 0.867 | 0.797 |  | 8.000 | 0.778 | 0.765 |
| *EWP* *WPF* QWV | | |  | *MIW IWE WED* | | |
| -1k9sAD- |  |  |  | -1fs5A- |  |  |
| 11.000 | 0.000 | 0.000 |  | 11.000 | 0.000 | 0.010 |
| 10.500 | 0.000 | 0.000 |  | 10.500 | 0.000 | 0.029 |
| 10.000 | 0.041 | 0.020 |  | 10.000 | 0.000 | 0.068 |
| 9.500 | 0.143 | 0.088 |  | 9.500 | 0.094 | 0.184 |
| 9.000 | 0.531 | 0.250 |  | 9.000 | 0.250 | 0.403 |
| 8.500 | 0.694 | 0.520 |  | 8.500 | 0.625 | 0.621 |
| 8.000 | 0.918 | 0.824 |  | 8.000 | 0.844 | 0.835 |
| HGM *NDM* MGH | | |  | *HMW MWT NHM* | | |

| **Scarcity score** | **TP** | **FP** |  | **Scarcity score** | **TP** | **FP** |
| --- | --- | --- | --- | --- | --- | --- |
| -1gai_- |  |  |  | -1n2uA- |  |  |
| 11.000 | 0.000 | 0.010 |  | 11.000 | 0.016 | 0.024 |
| 10.500 | 0.043 | 0.026 |  | 10.500 | 0.065 | 0.037 |
| 10.000 | 0.170 | 0.062 |  | 10.000 | 0.145 | 0.073 |
| 9.500 | 0.362 | 0.119 |  | 9.500 | 0.323 | 0.098 |
| 9.000 | 0.574 | 0.258 |  | 9.000 | 0.468 | 0.256 |
| 8.500 | 0.745 | 0.483 |  | 8.500 | 0.677 | 0.524 |
| 8.000 | 0.915 | 0.791 |  | 8.000 | 0.855 | 0.841 |
| *WCD* *SWC* *CSW* | | |  | *QWC WCD* PWM | | |
| -1ggf- |  |  |  | -1cml- |  |  |
| 11.000 | 0.000 | 0.007 |  | 11.000 | 0.000 | 0.000 |
| 10.500 | 0.000 | 0.025 |  | 10.500 | 0.000 | 0.014 |
| 10.000 | 0.022 | 0.044 |  | 10.000 | 0.000 | 0.058 |
| 9.500 | 0.130 | 0.115 |  | 9.500 | 0.000 | 0.140 |
| 9.000 | 0.370 | 0.275 |  | 9.000 | 0.200 | 0.270 |
| 8.500 | 0.717 | 0.543 |  | 8.500 | 0.200 | 0.471 |
| 8.000 | 0.902 | 0.863 |  | 8.000 | 0.800 | 0.799 |
| *FHW VMW MWA* | | |  | *MMY YMM YMY* | | |
| -1gm7AB- |  |  |  | -1eexABC- |  |  |
| 11.000 | 0.000 | 0.003 |  | 11.000 | 0.000 | 0.000 |
| 10.500 | 0.000 | 0.021 |  | 10.500 | 0.000 | 0.004 |
| 10.000 | 0.039 | 0.101 |  | 10.000 | 0.050 | 0.024 |
| 9.500 | 0.143 | 0.164 |  | 9.500 | 0.174 | 0.101 |
| 9.000 | 0.338 | 0.345 |  | 9.000 | 0.306 | 0.222 |
| 8.500 | 0.675 | 0.629 |  | 8.500 | 0.545 | 0.496 |
| 8.000 | 0.974 | 0.856 |  | 8.000 | 0.868 | 0.822 |
| *WDW* *WTH* *YWP* | | |  | *HMN HTC MMM* | | |
| -1gkm- |  |  |  | -2tlx_- |  |  |
| 11.000 | 0.000 | 0.002 |  | 11.000 | 0.000 | 0.000 |
| 10.500 | 0.000 | 0.007 |  | 10.500 | 0.022 | 0.000 |
| 10.000 | 0.000 | 0.038 |  | 10.000 | 0.065 | 0.030 |
| 9.500 | 0.000 | 0.080 |  | 9.500 | 0.152 | 0.105 |
| 9.000 | 0.071 | 0.194 |  | 9.000 | 0.478 | 0.304 |
| 8.500 | 0.357 | 0.390 |  | 8.500 | 0.609 | 0.612 |
| 8.000 | 0.571 | 0.709 |  | 8.000 | 0.848 | 0.869 |
| *WEM HMS HMS* | | |  | FWN AFW *HYY* | | |
| -1o6g- |  |  |  | -1eixAB- |  |  |
| 11.000 | 0.023 | 0.005 |  | 11.000 | 0.000 | 0.000 |
| 10.500 | 0.070 | 0.024 |  | 10.500 | 0.000 | 0.000 |
| 10.000 | 0.140 | 0.076 |  | 10.000 | 0.041 | 0.028 |
| 9.500 | 0.233 | 0.189 |  | 9.500 | 0.143 | 0.069 |
| 9.000 | 0.581 | 0.382 |  | 9.000 | 0.306 | 0.188 |
| 8.500 | 0.744 | 0.618 |  | 8.500 | 0.531 | 0.472 |
| 8.000 | 0.953 | 0.886 |  | 8.000 | 0.796 | 0.736 |
| *WYC WTH* HAW | | |  | *MMT QKC RMM* | | |

| **Scarcity score** | **TP** | **FP** |  | **Scarcity score** | **TP** | **FP** |
| --- | --- | --- | --- | --- | --- | --- |
| -1erm- |  |  |  | -1pnf_- |  |  |
| 11.000 | 0.000 | 0.000 |  | 11.000 | 0.107 | 0.000 |
| 10.500 | 0.037 | 0.011 |  | 10.500 | 0.143 | 0.012 |
| 10.000 | 0.130 | 0.023 |  | 10.000 | 0.250 | 0.055 |
| 9.500 | 0.167 | 0.068 |  | 9.500 | 0.500 | 0.109 |
| 9.000 | 0.352 | 0.210 |  | 9.000 | 0.714 | 0.313 |
| 8.500 | 0.500 | 0.432 |  | 8.500 | 0.821 | 0.633 |
| 8.000 | 0.833 | 0.778 |  | 8.000 | 1.000 | 0.902 |
| DWM *KHW* WME | | |  | EWC WCP GWC | | |
| -1hduA- |  |  |  | -1dz8A- |  |  |
| 11.000 | 0.000 | 0.000 |  | 11.000 | 0.000 | 0.008 |
| 10.500 | 0.000 | 0.000 |  | 10.500 | 0.000 | 0.023 |
| 10.000 | 0.067 | 0.047 |  | 10.000 | 0.054 | 0.071 |
| 9.500 | 0.133 | 0.142 |  | 9.500 | 0.141 | 0.173 |
| 9.000 | 0.400 | 0.362 |  | 9.000 | 0.370 | 0.301 |
| 8.500 | 0.644 | 0.629 |  | 8.500 | 0.576 | 0.586 |
| 8.000 | 0.822 | 0.866 |  | 8.000 | 0.815 | 0.872 |
| *WSY* RNW *WFA* | | |  | *CPM HWI PMH* | | |
| -1ma0_- |  |  |  | -1ibv- |  |  |
| 11.000 | 0.011 | 0.000 |  | 11.000 | 0.000 | 0.000 |
| 10.500 | 0.023 | 0.009 |  | 10.500 | 0.000 | 0.012 |
| 10.000 | 0.092 | 0.040 |  | 10.000 | 0.054 | 0.053 |
| 9.500 | 0.195 | 0.138 |  | 9.500 | 0.189 | 0.135 |
| 9.000 | 0.379 | 0.232 |  | 9.000 | 0.432 | 0.320 |
| 8.500 | 0.598 | 0.451 |  | 8.500 | 0.676 | 0.619 |
| 8.000 | 0.885 | 0.848 |  | 8.000 | 1.000 | 0.889 |
| HYM CHK *MGC* | | |  | *QWT QWD TQW* | | |
| -1mka- |  |  |  | -1cq1A- |  |  |
| 11.000 | 0.040 | 0.000 |  | 11.000 | 0.038 | 0.003 |
| 10.500 | 0.080 | 0.011 |  | 10.500 | 0.058 | 0.006 |
| 10.000 | 0.100 | 0.032 |  | 10.000 | 0.135 | 0.051 |
| 9.500 | 0.180 | 0.108 |  | 9.500 | 0.192 | 0.119 |
| 9.000 | 0.380 | 0.323 |  | 9.000 | 0.442 | 0.318 |
| 8.500 | 0.660 | 0.505 |  | 8.500 | 0.692 | 0.594 |
| 8.000 | 0.820 | 0.817 |  | 8.000 | 0.962 | 0.858 |
| HYM CHK *MGC* | | |  | CWP ICW *WPN* | | |
| -1o7n_- |  |  |  | -1e8gA- |  |  |
| 11.000 | 0.034 | 0.002 |  | 11.000 | 0.000 | 0.012 |
| 10.500 | 0.034 | 0.019 |  | 10.500 | 0.012 | 0.031 |
| 10.000 | 0.103 | 0.075 |  | 10.000 | 0.096 | 0.086 |
| 9.500 | 0.241 | 0.160 |  | 9.500 | 0.181 | 0.179 |
| 9.000 | 0.621 | 0.342 |  | 9.000 | 0.337 | 0.325 |
| 8.500 | 0.862 | 0.560 |  | 8.500 | 0.627 | 0.585 |
| 8.000 | 1.000 | 0.857 |  | 8.000 | 0.855 | 0.871 |
| WTH *WHT* *TWH* | | |  | *HWM WMM MMH* | | |

| **Scarcity score** | **TP** | **FP** |  | **Scarcity score** | **TP** | **FP** |
| --- | --- | --- | --- | --- | --- | --- |
| -1bd0- |  |  |  | -4reqAB- |  |  |
| 11.000 | 0.000 | 0.000 |  | 11.000 | 0.006 | 0.002 |
| 10.500 | 0.063 | 0.000 |  | 10.500 | 0.017 | 0.007 |
| 10.000 | 0.104 | 0.031 |  | 10.000 | 0.080 | 0.037 |
| 9.500 | 0.229 | 0.124 |  | 9.500 | 0.201 | 0.091 |
| 9.000 | 0.417 | 0.289 |  | 9.000 | 0.391 | 0.218 |
| 8.500 | 0.750 | 0.533 |  | 8.500 | 0.580 | 0.454 |
| 8.000 | 0.833 | 0.804 |  | 8.000 | 0.816 | 0.780 |
| CMD ICM DQC | | |  | *WHI* YHM *MDW* | | |
| -1g49B- |  |  |  | -2tsc- |  |  |
| 11.000 | 0.000 | 0.000 |  | 11.000 | 0.000 | 0.006 |
| 10.500 | 0.000 | 0.016 |  | 10.500 | 0.051 | 0.036 |
| 10.000 | 0.033 | 0.063 |  | 10.000 | 0.169 | 0.097 |
| 9.500 | 0.200 | 0.118 |  | 9.500 | 0.322 | 0.206 |
| 9.000 | 0.433 | 0.276 |  | 9.000 | 0.458 | 0.364 |
| 8.500 | 0.667 | 0.622 |  | 8.500 | 0.695 | 0.661 |
| 8.000 | 0.833 | 0.913 |  | 8.000 | 0.932 | 0.897 |
| *QWT PKW WTK* | | |  | *HMM HQM* PCH | | |
| -3apr_- |  |  |  | -1oneA- |  |  |
| 11.000 | 0.000 | 0.000 |  | 11.000 | 0.000 | 0.000 |
| 10.500 | 0.000 | 0.000 |  | 10.500 | 0.000 | 0.012 |
| 10.000 | 0.025 | 0.023 |  | 10.000 | 0.000 | 0.032 |
| 9.500 | 0.050 | 0.090 |  | 9.500 | 0.061 | 0.078 |
| 9.000 | 0.250 | 0.234 |  | 9.000 | 0.327 | 0.174 |
| 8.500 | 0.525 | 0.523 |  | 8.500 | 0.449 | 0.464 |
| 8.000 | 0.825 | 0.847 |  | 8.000 | 0.796 | 0.777 |
| *QCI* WSI *CTN* | | |  | *WSH KWM WMG* | | |
| -6cel- |  |  |  | -1ra2- |  |  |
| 11.000 | 0.022 | 0.000 |  | 11.000 | 0.027 | 0.000 |
| 10.500 | 0.045 | 0.006 |  | 10.500 | 0.027 | 0.019 |
| 10.000 | 0.129 | 0.047 |  | 10.000 | 0.108 | 0.115 |
| 9.500 | 0.270 | 0.141 |  | 9.500 | 0.203 | 0.250 |
| 9.000 | 0.489 | 0.253 |  | 9.000 | 0.378 | 0.385 |
| 8.500 | 0.764 | 0.582 |  | 8.500 | 0.527 | 0.673 |
| 8.000 | 0.927 | 0.876 |  | 8.000 | 0.797 | 0.846 |
| CDW WRW WTH | | |  | HTW MPW *YCF* | | |
| -1jkxA- |  |  |  | -1ldm- |  |  |
| 11.000 | 0.000 | 0.000 |  | 11.000 | 0.000 | 0.000 |
| 10.500 | 0.000 | 0.017 |  | 10.500 | 0.011 | 0.010 |
| 10.000 | 0.000 | 0.061 |  | 10.000 | 0.074 | 0.057 |
| 9.500 | 0.140 | 0.130 |  | 9.500 | 0.138 | 0.155 |
| 9.000 | 0.316 | 0.243 |  | 9.000 | 0.298 | 0.392 |
| 8.500 | 0.579 | 0.461 |  | 8.500 | 0.596 | 0.552 |
| 8.000 | 0.877 | 0.861 |  | 8.000 | 0.830 | 0.825 |
| *MHE KMH WFA* | | |  | *PMH* HGW *HSC* | | |

| **Scarcity score** | **TP** | **FP** |  | **Scarcity score** | **TP** | **FP** |
| --- | --- | --- | --- | --- | --- | --- |
| -1m7yA- |  |  |  | -1de6A- |  |  |
| 11.000 | 0.000 | 0.009 |  | 11.000 | 0.037 | 0.009 |
| 10.500 | 0.017 | 0.018 |  | 10.500 | 0.074 | 0.022 |
| 10.000 | 0.050 | 0.074 |  | 10.000 | 0.167 | 0.069 |
| 9.500 | 0.117 | 0.163 |  | 9.500 | 0.296 | 0.140 |
| 9.000 | 0.183 | 0.329 |  | 9.000 | 0.537 | 0.268 |
| 8.500 | 0.583 | 0.609 |  | 8.500 | 0.704 | 0.498 |
| 8.000 | 0.833 | 0.828 |  | 8.000 | 0.907 | 0.794 |
| *FCW CWV CHC* | | |  | HCW CWQ *MYC* | | |
| -1uae- |  |  |  | -1jcr_- |  |  |
| 11.000 | 0.000 | 0.003 |  | 11.000 | 0.037 | 0.007 |
| 10.500 | 0.000 | 0.013 |  | 10.500 | 0.083 | 0.033 |
| 10.000 | 0.066 | 0.032 |  | 10.000 | 0.241 | 0.078 |
| 9.500 | 0.131 | 0.086 |  | 9.500 | 0.352 | 0.159 |
| 9.000 | 0.311 | 0.210 |  | 9.000 | 0.565 | 0.313 |
| 8.500 | 0.525 | 0.429 |  | 8.500 | 0.750 | 0.565 |
| 8.000 | 0.885 | 0.781 |  | 8.000 | 0.944 | 0.832 |
| *IMC* *MCA* *DMH* | | |  | CYW *HWM* *MCC* | | |
| -1e9gA- |  |  |  | -1lee- |  |  |
| 11.000 | 0.000 | 0.000 |  | 11.000 | 0.000 | 0.000 |
| 10.500 | 0.000 | 0.015 |  | 10.500 | 0.018 | 0.008 |
| 10.000 | 0.083 | 0.078 |  | 10.000 | 0.073 | 0.038 |
| 9.500 | 0.146 | 0.121 |  | 9.500 | 0.109 | 0.121 |
| 9.000 | 0.375 | 0.262 |  | 9.000 | 0.218 | 0.339 |
| 8.500 | 0.604 | 0.534 |  | 8.500 | 0.418 | 0.594 |
| 8.000 | 0.854 | 0.893 |  | 8.000 | 0.855 | 0.874 |
| *WFF NCF QTW* | | |  | YWQ *MFY* *LCM* | | |
| -1oaf- |  |  |  | -1lmt_- |  |  |
| 11.000 | 0.000 | 0.000 |  | 11.000 | 0.074 | 0.022 |
| 10.500 | 0.027 | 0.000 |  | 10.500 | 0.074 | 0.022 |
| 10.000 | 0.080 | 0.000 |  | 10.000 | 0.148 | 0.174 |
| 9.500 | 0.133 | 0.043 |  | 9.500 | 0.222 | 0.283 |
| 9.000 | 0.227 | 0.213 |  | 9.000 | 0.481 | 0.478 |
| 8.500 | 0.533 | 0.511 |  | 8.500 | 0.815 | 0.685 |
| 8.000 | 0.813 | 0.780 |  | 8.000 | 1.000 | 0.826 |
| AWH WHS GPW | | |  | *WMC* YWC WCN | | |
| -2dhc- |  |  |  |  |  |  |
| 11.000 | 0.000 | 0.000 |  |  |  |  |
| 10.500 | 0.030 | 0.016 |  |  |  |  |
| 10.000 | 0.121 | 0.077 |  |  |  |  |
| 9.500 | 0.273 | 0.186 |  |  |  |  |
| 9.000 | 0.485 | 0.356 |  |  |  |  |
| 8.500 | 0.727 | 0.632 |  |  |  |  |
| 8.000 | 0.939 | 0.883 |  |  |  |  |
| WKY *WQN* *FWQ* | | |  |  | | |

(*) top ranking (highest scarcity score) 3-grams are listed in each case. The TPs and FPs are written in plain face and italic, respectively.

**B. Case studies.**

Tables S5 – S7 list the amino acids with the highest scarcity scores for the three respective test cases: Src kinase, hemoglobin, and tyrosyl tRNA synthetase. Conserved amino acids (entropy ≤ 7.5) are shaded in gray and fully conserved residues are shown in bold. Columns 1 and 2 give the residue index and type; column 3 gives the Shannon’s entropy[[1]](#footnote-2) defined as: -Σ*_i_ p_i_ ln p_i_*, were *p­_i_* is probability of occurrence of the *i*th amino acid at a given position in the aligned sequences and the summation is performed over all amino acids. Columns 4 and 5 give the average frequency and scarcity score (eq 5) based on UniProt distributions of 3-grams.

**Table S5: Src kinase amino acids distinguished by their high scarcity score**

| **Index** | **Amino Acid** | **Entropy** | **Average Frequency**  **<*p_j_^uni^*>** | **Scarcity Score**  **<*s_j_^uni^* >** |
| --- | --- | --- | --- | --- |
| 117 | D | 2.938 | 4.213E-05 | 10.075 |
| 118 | W | 1.557 | 3.950E-05 | 10.139 |
| 119 | W | 2.027 | 3.080E-05 | 10.388 |
| 120 | L | 2.955 | 3.506E-05 | 10.258 |
| 146 | E | 2.325 | 4.168E-05 | 10.086 |
| 147 | E | 2.505 | 3.182E-05 | 10.356 |
| 148 | W | 0.736 | 2.920E-05 | 10.441 |
| 149 | Y | 1.471 | 2.999E-05 | 10.415 |
| 150 | F | 1.648 | 3.142E-05 | 10.368 |
| 200 | K | 2.258 | 4.649E-05 | 9.976 |
| 319 | H | 0.075 | 5.102E-05 | 9.883 |
| 382 | Y | 1.586 | 4.938E-05 | 9.916 |
| 383 | V | 1.134 | 5.180E-05 | 9.868 |
| **384** | **H** | **0.000** | **5.170E-05** | **9.870** |
| 426 | I | 1.088 | 3.223E-05 | 10.343 |
| 427 | K | 0.726 | 3.066E-05 | 10.392 |
| **428** | **W** | **0.000** | **3.066E-05** | **10.393** |
| 429 | T | 0.739 | 3.198E-05 | 10.350 |
| 430 | A | 0.601 | 4.428E-05 | 10.025 |
| **444** | **D** | **0.000** | **5.189E-05** | **9.866** |
| 445 | V | 0.207 | 5.189E-05 | 9.866 |
| **446** | **W** | **0.000** | **3.400E-05** | **10.289** |
| 447 | S | 0.500 | 3.400E-05 | 10.289 |
| 448 | F | 0.726 | 3.400E-05 | 10.289 |
| 485 | P | 3.069 | 4.732E-05 | 9.959 |
| 487 | C | 0.619 | 5.194E-05 | 9.865 |
| 496 | C | 3.372 | 4.707E-05 | 9.964 |
| 497 | Q | 3.101 | 1.262E-05 | 11.280 |
| 498 | C | 0.257 | 9.617E-06 | 11.552 |
| 499 | W | 0.075 | 9.612E-06 | 11.553 |
| 500 | R | 2.813 | 1.154E-05 | 11.370 |
| 501 | K | 2.833 | 3.822E-05 | 10.172 |

**Table S6**

**(a) Hemoglobin subunit α residues distinguished by high scarcity score**

| **Index** | **Amino Acid** | **Entropy** | **Average Frequency**  **<*p_j_^uni^*>** | **Scarcity Score**  **<*s_j_^uni^* >** |
| --- | --- | --- | --- | --- |
| 12 | A | 1.486 | 6.309E-05 | 9.671 |
| 13 | A | 1.591 | 4.663E-05 | 9.973 |
| 14 | W | 0.227 | 4.230E-05 | 10.071 |
| 15 | G | 1.952 | 4.326E-05 | 10.048 |
| 16 | K | 0.301 | 4.757E-05 | 9.953 |
| **31** | **R** | **0.000** | **6.301E-05** | **9.672** |
| 32 | M | 0.904 | 5.581E-05 | 9.794 |
| **33** | **F** | **0.000** | **5.484E-05** | **9.811** |
| **42** | **Y** | **0.000** | **6.642E-05** | **9.619** |
| **43** | **F** | **0.000** | **5.039E-05** | **9.896** |
| 44 | P | 0.290 | 4.990E-05 | 9.906 |
| 45 | H | 0.063 | 4.959E-05 | 9.912 |
| 46 | F | 0.283 | 5.214E-05 | 9.862 |
| 47 | D | 0.153 | 6.927E-05 | 9.577 |
| **87** | **H** | **0.000** | **4.553E-05** | **9.997** |
| **88** | **A** | **0.000** | **4.553E-05** | **9.997** |
| 89 | H | 0.874 | 4.553E-05 | 9.997 |
| 102 | S | 0.360 | 4.140E-05 | 10.092 |
| 103 | H | 0.125 | 4.136E-05 | 10.093 |
| 104 | C | 0.637 | 4.174E-05 | 10.084 |
| 105 | L | 0.236 | 5.864E-05 | 9.744 |

**(b) Hemoglobin subunit β residues distinguished by their high scarcity score**

| **Index** | **Amino Acid** | **Entropy** | **Average Frequency**  **<*p_j_^uni^*>** | **Scarcity Score**  **<*s_j_^uni^* >** |
| --- | --- | --- | --- | --- |
| 15 | W | 0.110 | 4.473E-05 | 10.015 |
| 16 | G | 1.261 | 4.607E-05 | 9.985 |
| 17 | K | 0.133 | 4.685E-05 | 9.969 |
| 35 | Y | 0.044 | 2.207E-05 | 10.721 |
| **36** | **P** | **0.000** | **2.207E-05** | **10.721** |
| **37** | **W** | **0.000** | **2.205E-05** | **10.722** |
| **38** | **T** | **0.000** | **2.877E-05** | **10.456** |
| 39 | Q | 0.373 | 2.896E-05 | 10.450 |
| **91** | **L** | **0.000** | **4.636E-05** | **9.979** |
| **92** | **H** | **0.000** | **2.808E-05** | **10.480** |
| **93** | **C** | **0.000** | **2.808E-05** | **10.480** |
| 94 | D | 0.124 | 2.808E-05 | 10.480 |
| 143 | H | 0.423 | 4.204E-05 | 10.077 |
| 144 | K | 0.704 | 4.204E-05 | 10.077 |
| **145** | **Y** | **0.000** | **4.204E-05** | **10.077** |
| **146** | **H** | **0.000** | **4.712E-05** | **9.963** |

**Table S7: Tyrosyl tRNA synthetase amino acids distinguished by high scarcity score**

| **Index** | **Amino Acid** | **Entropy** | **Average Frequency**  **<*p_j_^uni^*>** | **Scarcity Score**  **<*s_j_^uni^* >** |
| --- | --- | --- | --- | --- |
| 124 | Y | 2.849 | 4.226E-05 | 10.072 |
| 125 | D | 2.411 | 3.454E-05 | 10.274 |
| **126** | **W** | **0.000** | **3.336E-05** | **10.308** |
| 127 | I | 2.236 | 4.539E-05 | 10.000 |
| 128 | G | 2.553 | 5.247E-05 | 9.855 |
| **194** | **D** | **0.000** | **3.556E-05** | **10.244** |
| **195** | **Q** | **0.000** | **3.511E-05** | **10.257** |
| 196 | W | 0.723 | 4.537E-05 | 10.001 |
| 197 | G | 0.454 | 4.584E-05 | 9.990 |
| **198** | **N** | **0.000** | **4.080E-05** | **10.107** |
| 252 | Y | 0.592 | 4.037E-05 | 10.117 |
| **253** | **Q** | **0.000** | **2.638E-05** | **10.543** |
| 254 | F | 0.985 | 2.825E-05 | 10.474 |
| 255 | W | 1.222 | 3.867E-05 | 10.161 |

Thus, 3-grams recruited at active sites are combinations of residues which already have relatively low natural frequencies of occurrence, in general. Yet, particular 3-grams exhibit in the UniProt significant departure from their expected occurrence probabilities as calculated using eq. 4. Table 5 lists the amino acids frequencies in the UniProt. Tables 6 and 7 list (in column 3) the most enhanced (*a_i_* > 1) and the least enhanced (*a_i_* < 1) *3*-grams that were extracted from the active sites in the Gutteridge and Thornton[[2]](#footnote-3) dataset of 59 enzymes.

**C**. **Scarcity scores: single sequence analysis vs. averaging over homologous sequences**

Here we show that functional sites can be identified even upon consideration of the scarcity score corresponding to only a single chain i.e. <*s_j_^uni^* >*= -* ln [*p_j_^uni^*], and we compare the results with those obtained by averaging the scarcity score over a set of homologous proteins (see eq. 5 manuscript).

The figure below displays the single chain scarcity scores for the α-subunit of hemoglobin. The resemblance of the results to those presented in Figure 7b (where average scores over a set of homologous proteins have been reported) indicates that scarcity and conservation are two different features.

**Figure 1S**

Hemoglobin chain α

Six stretches of amino acids are distinguished by their high scarcity scores: W_14_GK_16_, R_31_MF_33_, F_43_PHF_46_, D_75_MPN_78_, H_87_AH_89_, S_102_HCL_105_, in accord with the results presented in the manuscript (figure 7). The only stretch of amino acids that differs from the analysis by averaging over homologous proteins is D_75_MPN_78_.

Hemoglobin chain β

The single sequence analysis applied to hemoglobin β-subunit reveals five stretches of amino acids distinguished by their high scarcity scores: W_15_GK_17_, Y_35_PWTQ_39_, L_91_HCDK_95_, *A_115_HHF_118_*, H_143_KYH_146_. The only difference between these results obtained from single chain analysis and those found by averaging over homologous proteins is the occurrence of the A_115_HHF_118_ stretch of amino acids here. This stretch has not been implicated in any functional role, to our knowledge, and apparently the averaging over homologous sequences helps eliminate such single incidences from the results.

**Figure S2**

Src

The single sequence analysis for c-Src reveals ten stretches of amino acids distinguished by their high scarcity scores: D_117_WWL_120_, W_148_YF_150_, *A_183_YC_185_*, *C_238_HR_240_*, *W_260_EI_262_*, *V_281_WMG_284_*, I_426_KWT_429_, W_446_SF_448_, *M481PC483*, and L_494_MCQCWR_500_. Five of them correspond to stretches of amino acids that were revealed when averaging over homologous sequences and five do not (shown in italic). **For two of the five newly detected stretches two have functional roles. W_260_EI_262_ includes the conserved tryptophan W260, at the C-terminus of the SH2 - catalytic domain linker. Gonfloni *et al* {Gonfloni, 1997 9 /id} showed that the W260A mutation impaired the regulation of C-Src in yeast; similarly LaFèvre-Bernt *et al*{LaFevre-Bernt, 1998 10 /id} concluded that in the inactive conformation W260 holds the αC helix and Glu310 in an inactive position and plays a role in positioning the SH2 and SH3 domains for intra- and intermolecular interactions. The second stretch of interest,** V_281_WMG_284_**,** corresponds to the ATP binding site.

**Figure S3**

Tyrosyl-tRNA synthetase

The single sequence analysis in the case of tyrosyl-tRNA synthetase yields the stretches of amino acids Y_124_DW_126_, *Y_147_MM_149_*, *Y_169_MM_171_*, D_194_QWG_197_, Q_253_FW_255_. Two stretches of amino acids (shown in italic) are detected using the single sequence analysis but not when averaging over homologous sequences. One of them, *Y_147_MM_149_* has unknown functional role, the other *Y_169_MM_171_* corresponds to the substrate binding site.

Thus averaging over homologous sequences appears to eliminate some false positives, while some true positives may be overlooked if the particular signal is unique to the examined sequence only, and not shared by the members of the same family.

1. Shannon,C.E. (1948) The mathematical theory of communication. *The Bell system Technical Journal*, 27, 379–423 and 623–656. [↑](#footnote-ref-2)
2. Gutteridge,A. and Thornton,J. (2005) Conformational changes observed in enzyme crystal structures upon substrate binding. *J. Mol. Biol*, 346, 21-28. [↑](#footnote-ref-3)
